# Different forms of effective connectivity in primate frontotemporal pathways

**DOI:** 10.1038/ncomms7000

**Published:** 2015-01-23

**Authors:** Christopher I. Petkov, Yukiko Kikuchi, Alice E. Milne, Mortimer Mishkin, Josef P. Rauschecker, Nikos K. Logothetis

**Affiliations:** 1 Institute of Neuroscience, Framlington Place, Newcastle University, Newcastle upon Tyne NE2 4HH, UK; 2 Department of Physiology of Cognitive Processes, Max Planck Institute for Biological Cybernetics, 38 Spemannstrasse, 72076 Tübingen, Germany; 3 Laboratory of Neuropsychology, NIMH, NIH, 31 Center Drive, Bethesda, Maryland 20892, USA; 4 Department of Neuroscience, Georgetown University Medical Center, 3970 Reservoir Road, N.W., Washington, District of Columbia 20057, USA; 5 Division of Imaging Science and Biomedical Engineering, University of Manchester, Stopford Building, Oxford Road, Manchester M13 9PT, UK; 6 Institute for Advanced Study, Technische Universität München, Lichtenbergstrasse 2a, Garching 85748, Germany

## Abstract

It is generally held that non-primary sensory regions of the brain have a strong impact on frontal cortex. However, the effective connectivity of pathways to frontal cortex is poorly understood. Here we microstimulate sites in the superior temporal and ventral frontal cortex of monkeys and use functional magnetic resonance imaging to evaluate the functional activity resulting from the stimulation of interconnected regions. Surprisingly, we find that, although certain earlier stages of auditory cortical processing can strongly activate frontal cortex, downstream auditory regions, such as voice-sensitive cortex, appear to functionally engage primarily an ipsilateral temporal lobe network. Stimulating other sites within this activated temporal lobe network shows strong activation of frontal cortex. The results indicate that the relative stage of sensory processing does not predict the level of functional access to the frontal lobes. Rather, certain brain regions engage local networks, only parts of which have a strong functional impact on frontal cortex.

## Abstract (web summary)

Neural pathways to frontal cortex areas of the brain enable communication, but their connectivity is unclear. Petkov *et al*. use electrical microstimulation and brain imaging to describe different forms of hierarchical effective connectivity that exist between the primate frontal and temporal cortex.

## Graphical abstract

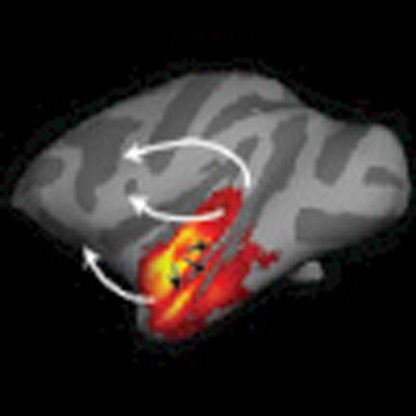

There is considerable interest in understanding the functional connectivity of the brain, including pathways to frontal cortex that enable communication^1^,^2^,^3^. Primary cortical areas, which are the sensory input recipients of the neocortex, are not as strongly interconnected with frontal cortex as are non-primary sensory and association areas^4^,^5^,^6^,^7^,^8^. Thereby, the generally held notion is that certain processing stages, by virtue of their position, have privileged access to frontal cortex. However, although there is substantial evidence that the brain processes information both in serial and parallel^7^,^9^, it is less clear whether there is a principal form of arrangement^10^,^11^. Since the effective functional connectivity of the brain is poorly understood, it remains possible that certain sensory processing stages, regardless of their position, are effective in engaging frontal cortex by themselves or interact with other regions to indirectly gain functional access to frontal cortex.

In the auditory system, primary auditory cortex has a local interconnectivity pattern, where neuronal connectivity is stronger between adjacent than non-adjacent cortical areas^12^. However, structures as early as the second stage of auditory cortical processing show direct projections to frontal cortex. Notably, tracer injections into the lateral auditory belt^9^,^10^ monosynaptically label axonal boutons in either ventral or dorsal frontal cortex^5^, depending on whether the injections are made in ventral or dorsal parts of the auditory belt^5^. These observations are consistent with the guiding notion of parallel processing pathways to frontal cortex^1^,^13^, where ventral and dorsal streams process object features and spatial content, respectively. Downstream from the auditory belt are anatomical areas Ts1/Ts2 (ref. 14), which represent the fourth or fifth anatomically defined stage of processing (Fig. 1a). This anterior/ventral temporal lobe region is known in humans^15^ and macaque monkeys^16^ to contain clusters of neurons^17^ sensitive to voice content in communication sounds, such as the acoustical features associated with voice identity, that is, ‘who’ vocalized. The functional characteristics of voice-sensitive neurons (which in macaques are located in the anterior supratemporal plane, aSTP; Fig. 1a) differ from those of neurons in the adjacent multisensory association cortex, in the upper bank of the anterior superior temporal sulcus (aSTS). Namely, aSTP neurons are more auditory feature sensitive and show less specific multisensory responses than neurons in the aSTS^18^; also see refs 19, 20, 21.

Given that the auditory belt projects to frontal cortex, one might expect neurons in aSTP areas Ts1/Ts2 to do the same. However, this remains unresolved. A number of earlier studies injecting anterograde tracers into Ts1/Ts2 report strong labelling in frontal cortex, but these studies also intended to make their tracer injections large enough to involve adjacent association cortex on the gyrus and aSTS, which is known to project to frontal cortex^6^,^22^,^23^. Others studying retrograde projections from ventral, orbital or medial frontal cortex to Ts1/Ts2 (or RTp by other nomenclature^24^) show a divergence of results, with some reporting strong^8^,^25^,^26^ and others negligible^27^,^28^ labelling. Nonetheless, some synapses could be more effective than others, and it has been difficult to address the direction of effective connectivity with the available neuroimaging approaches^4^,^29^. Therefore, the key question is: would a downstream sensory processing stage, such as voice-identity sensitive cortex in the aSTP, directly engage ventral frontal and orbital frontal cortex or would it interact with a local temporal lobe network to gain functional access to frontal cortex?

To tackle this question, we combined microstimulation and functional magnetic resonance imaging (fMRI) in rhesus macaques^30^,^31^,^32^,^33^,^34^,^35^. Neuronal microstimulation of a given cortical site elicits an fMRI response in interconnected regions but appears to be prevented from propagating loosely throughout the cortex by intracortical inhibition in the target regions^30^. In this case, it is possible that combined microstimulation and fMRI could be useful for charting the effective connectivity of a neuronal network, whereby after microstimulating a site and using fMRI to identify its activated targets, one of the demonstrated target sites could then be stimulated to reveal which additional areas become activated. Alternatively, in the case of less selective effects of combined microstimulation, one might expect stimulation of two adjacent regions to show indistinguishable fMRI activity patterns. Figure 1b,c illustrates two alternative hypotheses. In one case, aSTP stimulation mainly results in temporal lobe activity, whereas aSTS stimulation leads to additional frontal activation (Fig. 1b). Alternatively, stimulating the aSTP results in ventral and orbital/medial frontal activity that cannot be distinguished from that of stimulating the aSTS (which is known to project to orbital/medial frontal cortex^8^,^23^,^27^; see Fig. 1c). Note that in both cases, control experiments are needed to clarify the extent to which microstimulation and fMRI is consistent with established anterograde neuronal tractography results. This is illustrated in both Fig. 1b,c as anterior lateral belt fields projecting to ventral frontal cortex and more caudal lateral belt fields projecting to more dorsal parts of lateral frontal cortex^5^,^25^,^36^. Testing these hypotheses could reveal how auditory temporal lobe regions are connected with frontal cortex, and, with the auditory system as a model, our study could clarify whether there might be other exceptions to a principal form of cortical arrangement besides those coming from the visual system^7^,^10^,^11^.

Here we microstimulate fMRI-identified voice-sensitive clusters, localized to the aSTP and observe an fMRI response restricted to the ipsilateral anterior temporal lobe. By contrast, on stimulating another site within this activated network, a region in the aSTS, we additionally observe strong orbital frontal activation. Further experiments stimulating different parts of the auditory belt and the ventral frontal cortex confirm key prior neuronal anterograde tractography findings, clarifying the interpretation of the aSTP stimulation results. We provide evidence for different forms of effective connectivity in primate auditory temporal to frontal pathways, showing that certain non-primary auditory regions, like the voice-identity sensitive cortex in the aSTP, appear to rely on adjacent temporal lobe processes before gaining access to frontal cortex. This finding is in stark contrast to the results obtained by stimulating the auditory lateral belt and aSTS sites. Together the results raise the possibility that the auditory stage of processing does not predict the level of engagement of the frontal cortex.

## Results

### fMRI localizer results and microstimulation approach

In four rhesus macaques, we first used fMRI and sounds varying in frequency to localize the tonotopically organized auditory core and belt fields^37^. We also used fMRI to localize voice-sensitive clusters in the aSTP(in anatomical areas Ts1/Ts2 (refs 14, 16)). This region is anterior to the tonotopically organized auditory core (primary) and belt (secondary) fields and is known to be voice-identity sensitive^16^ (Fig. 2, Supplementary Figs 1–4). FMRI voice-area localization was conducted using voice versus non-voice or voice-identity adaptation experiments^16^. Our hypotheses focus on the Ts1/Ts2 regions; thus, voice-sensitive clusters were analysed only within these regions, although it is known that there are other voice-sensitive clusters in the human^15^ and macaque^16^ brain. Given the large number of localizers and experiments conducted here, all the fMRI localizer and microstimulation experiments were conducted under anaesthesia using an established protocol. The results from using this protocol have previously been compared and noted to be largely comparable to those obtained in awake animals^16^,^30^,^31^,^37^,^38^,^39^ (also see Supplementary Text and Discussion).

Voice-sensitive clusters in the aSTP were observed in the left or both hemispheres of the four study animals (Fig. 2, Supplementary Figs 1–4). This result is consistent with previous observations of a lack of significant lateralization of monkey voice clusters^16^. The first monkey (M1) had bilaterally distributed anterior voice-sensitive clusters (Supplementary Figs 1,5). Thus, in this animal, we implanted chambers over both hemispheres and compared the results of stimulating left- and right-hemisphere sites. We noted no qualitative hemispheric differences in several of the key findings reported here (Supplementary Fig. 5). Thus, the remaining animals (M2–4) were implanted with left-hemisphere chambers for consistency. In targeting fMRI-identified sites for microstimulation, we used the coordinates from the fMRI voice or tonotopy localizers. For structural MRI-identified sites, we used the coordinates of the MRI structural scans, which were referenced to a rhesus macaque brain atlas in stereotactic coordinates^24^. As the electrode was advanced to the target region, it generated a local MRI signal dropout, such that its general location could be identified in structural scans (Fig. 2 and Supplementary Figs 1–6). For greater targeting precision, as we slowly approached the target site, we monitored neuronal spiking activity relative to the neurophysiologically ‘quiet’ transition areas, such as the lateral sulcus above the aSTP or the white matter between the aSTP and aSTS. This allowed us to advance the electrode to be ~1 mm within the grey matter of the target site.

### Effects of microstimulating aSTP and aSTS sites

Microstimulation of both aSTP and aSTS sites was successful in three out of the four macaques studied (M1–3). In the fourth macaque (M4), the results from stimulating the aSTS were consistent with those from the other three macaques (Supplementary Fig. 7). However, stimulating the aSTP site in M4 did not result in significantly activated voxels anywhere in the brain (cluster corrected *P<*0.05). Therefore, only the aSTP and aSTS results available from M1–3 could be analytically compared and analysed further.

Microstimulation of the aSTP site in the three animals (M1–3; Figs 3, 4, 5) resulted in significant (cluster corrected *P<*0.05) fMRI blood oxygen level dependant (BOLD) responses from a number of regions, largely restricted to the ipsilateral anterior temporal lobe (Figs 3, 4, 5a). Notably, no significant contralateral (right) hemisphere activity was observed in any of the animals. The activated anatomical areas in common across the three animals, resulting from aSTP microstimulation, involved the aSTS, temporopolar cortex and anterior auditory cortical fields (ACFs; see Supplementary Fig. 8). Supplementary Tables 1–3 summarize the significantly activated anatomical areas seen in each animal. In two out of these three animals’ results, the following additional areas in the ipsilateral anterior temporal lobe and operculum were activated in common: visual area TE, agranular insula in the temporal operculum and area PrCO in the frontal operculum. Notably, there was no clear functional engagement of orbital, medial, dorsal or ventral frontal cortex.

Thereby, the results from microstimulating the voice-sensitive cluster in the aSTP showed clear functional activation of an anterior temporal network (including the aSTS) but not of the frontal cortex, barring area PrCO in the frontal operculum that was observed in the majority of stimulation cases. Because such a result may be due to the inherent limitations of direct electrical stimulation (DES)^40^ rather than to interarea connectivity, we tested whether stimulation of a target region within the observed activated local network would activate frontal cortex more strongly. We selected an aSTS site that we had observed was consistently activated by stimulating the aSTP (Figs 3, 4, 5a). Microstimulating the aSTS resulted in a significant activity response in a comparable ipsilateral anterior temporal lobe network, as seen by stimulating the aSTP. In sharp contrast, however, aSTS microstimulation prominently activated the orbital frontal cortex (OFC; Figs 3, 4, 5b; Supplementary Tables 1–3). The following anatomical areas were significantly activated in common in M1–3: adjacent aSTS regions, temporopolar cortex, anterior ACFs, visual area TE, hippocampus and entorhinal cortex and notably the orbital frontal cortex (area 13). In two of the three animals the additionally activated areas included the amygdala, agranular insula and the frontal operculum (area PrCo). Other results recapitulated these main observations and confirmed the reliability of the findings: See the further replication experiments from stimulating the left and right aSTP in M1 (Supplementary Figs 5 and 8), the right aSTP and aSTS in M1 (Supplementary Fig. 6) and stimulating the left aSTS in M4 (Supplementary Fig. 7).

For a more direct comparison between the effects of stimulating aSTS versus aSTP, we next analytically contrasted these results and also performed planned region-of-interest (ROI) analyses. The analytical comparison of aSTS versus aSTP in M1–3 showed significantly greater activity in the ipsilateral orbital frontal cortex and certain anterior temporal lobe sites, such as visual area TE (Figs 3, 4, 5c; thresholded at *P<*0.001 for consistency across the animals, see Methods; also see Supplementary Tables 1–3 for cluster voxel numbers and peak voxel *z* scores). Furthermore, an anatomically defined ROI analysis confirmed the stronger OFC activation by aSTS stimulation in the three animals (all results *P<*0.001; bar graphs in Figs 3, 4, 5c). We then combined the three monkeys’ results into a mixed-effects analysis of variance (ANOVA), with ‘monkey’ as a random between-subjects factor and ‘site’ of stimulation (aSTS or aSTP) as a within-subjects fixed factor. The result of this analysis recapitulated that stimulating the aSTS elicited greater activity in the OFC ROI than that produced by aSTP stimulation (F_1,752_=20.4, *P*=0.045). There was no significant effect of the monkey factor or interaction between the factors. When hemisphere was added to the model a significant effect of hemisphere was observed (F_1,1964_=62.81, *P*=0.015), confirming that the results were lateralized to the stimulated ipsilateral hemisphere. As a point of reference, analysing the effect of site of stimulation in an anterior auditory cortex ROI (consisting of anterior auditory core and belt areas^24^) showed no consistent effect of site of stimulation in the animals individually (Supplementary Fig. 9), suggesting that no over or underactivation of the anterior auditory cortex occurs with either aSTS or aSTP stimulation. This observation was recapitulated by conducting the combined animal ANOVA with the voxel-based responses from the anterior auditory cortex ROI, which showed no significant differences between aSTS or aSTP stimulation in the activation of anterior auditory core and belt areas.

In summary, the results showed greater OFC activation from the stimulation of the aSTS than from the stimulation of the aSTP. The aSTP stimulation did not strongly activate any frontal region, excepting the frontal operculum (area PrCO), which stimulation of either aSTP or aSTS could activate in the majority of cases. Both stimulation sites also resulted in strong ipsilateral activity involving the anterior temporal lobe. This reveals a common functional network, with the key difference that stimulating the aSTS (anatomical area TPO) resulted in orbital frontal activation. To understand these results in a broader context, we conducted further experiments in two of the macaques (M3–4) stimulating different sites in fMRI-identified tonotopically organized auditory belt fields and separately also in the ventral frontal cortex.

### Microstimulation of auditory belt fields

Romanski *et al*.^5^,^36^ injected anterograde tracers in the ventral/anterior and dorsal/caudal lateral belt (auditory cortical fields: AL and CL, respectively). They obtained results consistent with a dual pathways model^1^,^13^ and evidence that neurons as early as those in the belt, the second key stage of auditory cortical processing, project to frontal cortex^36^. They also noted that caudal auditory belt regions project to more dorsal regions in the lateral frontal cortex, whereas the more anterior regions in auditory cortex target ventral regions in the lateral and orbital frontal cortex^5^,^36^. Using fMRI tonotopic mapping^37^ we targeted for microstimulation field RTL, the most anterior lateral belt field situated in front of AL (Fig. 6). In another experiment, the placement of the chamber enabled targeting of ML, a mid/caudal lateral belt field (Fig. 7). Overall, our microstimulation results were complementary to those obtained by neuronal tractography studies^5^,^41^. First, unlike the results from stimulating aSTP and aSTS (Figs 3, 4, 5), stimulating these auditory belt fields resulted in significant (cluster corrected *P<*0.05) cross-hemisphere activation (including activation of homotopic auditory cortical fields in the opposite hemisphere; Supplementary Tables 4 and 5; Figs 6 and 7). This observation is consistent with the reported transcallosal tractography of auditory cortex^41^. Second, stimulating the auditory lateral belt resulted in strong frontal cortex activity. In the case of stimulating the mid/caudal belt field ML, activation was seen in more dorsal frontal areas 46 and area 8 (Fig. 7, Supplementary Table 5), which is generally consistent with the reported anterograde projection to frontal cortex of caudal belt field CL^5^,^36^. By comparison, stimulating the anterior belt field RTL resulted in more ventral frontal and orbital frontal (area 13) activity (Fig. 6, Supplementary Table 4), which is consistent with the projection pattern to frontal cortex of the anterior belt field AL^5^,^36^. Thus, the results from electrically stimulating lateral auditory belt fields correspond in a number of ways to the key findings from neuronal tracing studies, further underscoring the very different pattern of results seen from stimulating the aSTP and aSTS.

### Microstimulation of sites in ventral frontal cortex

A number of neuroanatomical studies have examined connections between frontal and temporal cortex^5^,^6^,^8^,^22^,^23^,^25^,^27^,^42^. The general observations from these studies is that dorsal parts of the frontal cortex interconnect with dorsal frontal, parietal and temporal areas, whereas more ventral frontal cortical areas are interconnected with ventral/anterior areas using ventral pathways such as the uncinate fasciculus or extreme capsule. In M3–4, we stimulated three areas in the ventral frontal cortex, differing along the dorsoventral axis (Figs 8, 9, 10). The results are consistent with the evidence that more dorsal parts of ventral frontal cortex are interconnected with more dorsal regions of the brain (Fig. 8). Specifically, stimulating the more dorsal of these frontal sites (area 45) produced the clearest contralateral engagement in these three experiments and resulted in significant activation (cluster corrected *P<*0.05) of relatively more dorsal regions such as areas 6, 8, 9 and 4 (Fig. 8; Supplementary Table 6). By contrast, stimulating a more ventral site in area 6va or area F5 resulted in more ventral regions being significantly activated, which included the frontal operculum, area 44/45, STS and auditory belt/parabelt. In this case, no significant activation is seen in dorsolateral frontal regions or in aSTP fields Ts1/Ts2 (Fig. 9, Supplementary Table 7). Stimulating the most ventral site (of the three shown in Fig. 10) within the operculum, near the border of the agranular and dysgranular insula, resulted in a strong activation of the anterior temporal lobe (including aSTP areas Ts1/Ts2 and the aSTS) and the adjacent orbital frontal cortex, among other ventral regions (Supplementary Table 8).

## Discussion

The results, obtained using combined microstimulation and fMRI to chart primate frontotemporal effective connectivity, challenge the notion that all non-primary brain regions have a strong functional impact on the frontal lobe. We observed that stimulation of certain earlier auditory processing regions, such as the lateral auditory belt, can significantly activate frontal cortex, consistent with neuroanatomical tracer studies^5^,^8^,^36^. However, stimulating further downstream auditory stages, such as voice-sensitive cortex in anatomical areas Ts1/Ts2 is seen to engage primarily an ipsilateral anterior temporal lobe network, only parts of which activate frontal cortex when stimulated. This study, therefore, supports notions of alternative arrangements of parallel processing streams^4^,^7^,^10^,^11^, suggesting how these can occur in the primate ventral auditory processing stream. We discuss below how the results provide an important effective connectivity perspective that is informed by neuronal tractography and can potentially disambiguate neuroimaging findings, which are often obtained from bidirectional connectivity data^29^.

In relation to prior neuronal tractography studies, our results reveal and demonstrate an interesting paradox in auditory temporal to frontal connectivity. The observation from neuronal tractography studies^5^ and our effective connectivity results (Figs 6 and 7) clearly show that auditory belt areas target frontal cortex. Thus, auditory cortical processing as early as the second stage has a direct functional impact on, at least, ventro- and dorsolateral frontal cortex, and our combined microstimulation and fMRI results from the lateral belt confirm these general findings. The unresolved question was whether all non-primary auditory regions would affect frontal cortex potentially more strongly than would early auditory areas^8^,^25^,^27^. Here we show that a downstream fMRI-identified voice-sensitive cluster in the aSTP (located within anatomical areas Ts1/Ts2, which represent the fourth or fifth stage of auditory cortical processing) does not prominently activate frontal cortex when stimulated. Notably, only by stimulating an identified functional target of this region in the adjacent multisensory aSTS did we observe prominent frontal activation involving, in particular, orbital frontal cortex. Given that both the aSTS and frontal cortex are considered to be at a higher processing level than the auditory sites that we stimulated, that is, aSTP or lateral belt), our results reveal that certain processes, such as those in the auditory lateral belt, can gain functional access to frontal cortex, while others, such as those in the anterior voice-sensitive cortex, engage a more local multisensory network before gaining access to orbital frontal cortex.

Classically, the Ts1/Ts2 anatomical region was considered as association (multisensory) cortex. However, at least parts of these anatomical areas on the aSTP are now known to contain a voice-sensitive region. This region appears to preferentially respond to voice-identity content in communication sounds^15^,^16^,^17^,^18^,^43^, although other voice-preferring clusters have also been identified with fMRI in the human and monkey brain^15^,^16^,^44^. Neurophysiological study of the anterior voice-sensitive region in monkeys shows that its constituent neurons are sensitive to different types of auditory inputs generally^17^,^21^ and differ in their functional characteristics from neurons in the adjacent association cortex of the aSTS. The aSTS, by comparison, is less auditory feature sensitive and shows greater specificity in multisensory influences^18^ (also see refs 19, 20, 21, 45). Thereby, these prior observations in the context of the current findings would suggest that the orbital frontal targets of the aSTS receive multisensory input from parts of an anterior temporal network, and that the aSTP interacts with this network to indirectly gain functional access to frontal cortex.

Neuronal tractography and computational studies in macaques^7^,^10^,^11^ and recent analyses of neuroimaging connectivity data in humans, macaques and other animals have noted exceptions to a unique parallel processing organization^4^, stemming primarily from work in the visual system. For example, the visual frontal eye fields appear to be an exception in the visual processing hierarchy in that they send strong feedforward laminar projections to dorsal stream visual areas in the temporoparietal cortex^11^. This is unusual as the frontal areas tend to provide feedback projections to upstream visual processing areas. As other examples, recent analyses of human and monkey connectivity data suggest that local clusters of processing are the rule rather than the exception, and that certain brain regions act as hubs with longer-range projections that interconnect different clusters. Our results suggest that association cortex in the aSTS is likely to be an important temporal lobe site for the access to frontal cortex. However, possibly because many of the neuroimaging approaches are based on bidirectional connectivity data, the results are not always consistent with known neuronal tractography connectivity patterns. For example, in some neuroimaging results human primary auditory cortex is seen to have some of the longest range projections, longer than association cortex^46^. Moreover, connectivity patterns in diffusion-weighted imaging data from humans and monkeys showing the clearest connectivity patterns appear to involve the dorsal frontoparietal and frontotemporal pathways^47^. By comparison, the ventral frontotemporal pathways are more difficult to delineate, in part because of crossing fibers in the uncinate fasciculus and extreme capsule.

The current study is an important complement to neuroimaging and neuroanatomical/neurophysiological work, providing insights on effective connectivity. However, neuroimaging-based approaches, ours included, do not have the specificity of neuronal tractography studies that can evaluate laminar projections to identify feedforward and feedback projections, and thus inform us on neuroanatomical hierarchies. Nonetheless, as we see in our study and others have noted for the visual system^48^, anatomically defined hierarchies need not be correlated with neuroimaging or neurophysiologically based topographies, the latter of which can be used to delineate the level of functional processing complexity. Our study was informed by work identifying the neurophysiological and neuroanatomical processing stages of the auditory lateral belt^5^,^8^,^13^,^25^,^49^,^50^ and information on voice-sensitive cortex in the macaque Ts1/Ts2 regions^14^,^16^,^17^,^18^,^22^,^51^. Also, our ‘control’ experiments stimulating the aSTS, two fields in the lateral belt and three in the frontal cortex, are remarkable in that, any limitations of DES and fMRI notwithstanding, they seem to recapitulate the key findings from anterograde neuronal tractography findings, as we have noted above. Thus, although an anatomically based hierarchy would place the aSTP as an anatomical stage in between the lateral belt and aSTS, our results show instead that aSTP stimulation does not prominently activate the frontal cortex (apart from the operculum in the majority of cases), whereas microstimulating the lateral belt and aSTS does result in significant activity of the orbital/ventral frontal cortex.

It is important to consider to what extent the results can and cannot account for alternative explanations, especially since the approach of combined DES and fMRI is not yet well understood (for reviews, see refs 40, 52). Trivial explanations cannot easily account for the differences in aSTP versus aSTS activation of the OFC. For example, the observation of greater OFC activity by aSTS but not by aSTP stimulation was supported by whole-brain and hypothesis-driven ROI analyses (the ROI were conducted at the individual and group levels), thus the main results seem to be statistically robust. Also stimulation trial numbers were matched between aSTS and aSTP comparisons, the stimulation current was fixed, and the animals were anesthetized (so that drifts in attention or any task-dependent effects would not contribute, both of which are known to be able to influence the magnitude of fMRI effects from DES^32^). The effects of stimulating the frontal eye fields, which reproduces some of the effects of attention, can differ depending on the locations activated relative to bottom-up influences from visual input^33^. Thus, given that stimulus and task-dependent effects might affect the findings sufficiently, and these effects also likely differ by site stimulated, studying effective connectivity in anesthetized animals seemed warranted as a first approach. It remains an interesting open possibility that presenting communication sounds varying in voice-identity content, with or without an active task, might have elicited stronger activity in frontal cortex in combination with microstimulation of the aSTP.

Any choice of anaesthesia could affect the BOLD response, although it seems unlikely that the anaesthesia protocol differently affected the aSTP and aSTS sites, both of which are separated by a few millimetres of white matter. Also, the aSTS site elicited robust activity in the OFC, so it is certainly the case that stimulating specific temporal lobe sites (lateral belt included) could robustly activate parts of the frontal cortex. Our anaesthesia protocol using remifentanil has been developed to minimally affect regions such as the ones reported here. Moreover, all of the approaches that we used for fMRI localization or microstimulation in anesthetized animals have been compared with the results obtained from awake animals and shown to be largely comparable to a number of different visual and auditory processes^16^,^30^,^31^,^37^,^38^,^39^ (for additional discussion of the impact of the anaesthetic on the BOLD response see the Supplementary Discussion).

Other alternative, not necessarily mutually exclusive, interpretations either find little support or cannot be excluded by our results. For example, is it the case that more robust activation of the aSTP might engage OFC, comparably to what we see with the aSTS? Our data do not appear to provide much support for this possibility, since the most statistically robust aSTS versus aSTP microstimulation result in activating OFC and MPFC that we obtained coincides with the most robust activation of the aSTP (Supplementary Fig. 6). However, on the question of whether other regions might need to be co-activated along with the aSTS to cause an effect in OFC, this remains an interesting possibility. The main region that was consistently recruited with aSTS stimulation but not aSTP stimulation was the OFC. However, a number of anterior temporal lobe regions were more strongly activated by aSTS than aSTP stimulation, many of which are known to project to frontal cortex (Figs 3, 4, 5; Supplementary Tables 1–3). Thus, although aSTP stimulation can significantly activate these same anterior temporal lobe regions, it remains possible that stronger activation of these regions by aSTS stimulation contributed to the stronger OFC activity seen. Neuronal tractography results show anterograde projections from the aSTS to the OFC, thus the elicited OFC activity might not depend on the co-activation of other regions. However, the possibility that co-activation of certain regions is required cannot be fully excluded without, for example, inactivating these said regions and seeing whether microstimulation of the aSTS would still activate the OFC.

Microstimulation and fMRI enabled us to chart the effective connectivity of a number of brain regions, in a way that, to our knowledge, has not been done before. For instance, we first identified the functional targets of a site, some of which were then stimulated to identify which new regions were significantly more activated by stimulating the demonstrated targets of a particular site. This approach aimed to harness what has otherwise been noted as a limitation of DES, that is, what appears to be relatively more restricted rather than extensive synaptic propagation^40^ (also see refs 32, 33, 34, 35). Namely, with DES there is evidence that gamma-aminobutyric acid (GABA)-ergic intracortical inhibition in the target region prevents the activity response from loosely propagating to other cortical afferents^30^. However, direct connections should not be assumed because corticosubcortical–cortical activation and antidromic activation remain possible and should be considered when interpreting the results. Nonetheless, our use of ‘effective connectivity’ is in line with the original definition, as a measure of the impact of one neural system on another either directly or indirectly^29^. This is distinguished from undirected or bidirectional functional connectivity.

Studies of combined microstimulation and fMRI, ours included, cannot precisely localize the stimulating electrode to a particular cortical layer. Also, current-spread measurements (in our case at least 0.62 mm radius^31^) and prior work with DES indicate that the most excitable (pyramidal) cells in the middle layers of the cortex are stimulated^53^. Optogenetic techniques enable greater selectively in optically simulating specific neuronal subgroups, which are ones that express genetically transfected channel rhodopsins. However, such an approach is also likely to engage intracortical inhibition in the target site, which although limiting loose transcortical propagation^54^, appeared to be an advantage in this study. Also, at least currently, combined optogenetic and fMRI studies in monkeys require cell-nonspecific genetic promotors^55^ to elicit robust-enough neuronal responses in primates that can be measured with fMRI. Thus, similar to microstimulation, only the most excitable cells in the cortex would be optogenetically stimulated. The limitations of the approach notwithstanding, we included several control stimulation experiments that recapitulated a number of key established findings from the neuronal tractography literature. This provides an important point of reference and helps to interpret the results of microstimulating the aSTP. Other studies using combined DES and fMRI of cortical regions in the somatosensory^35^ or visual system^32^,^33^,^34^ have also reported that a number of their results are consistent with the prominent projection patterns reported in neuronal tractography studies. All in all, our results suggest that the reported approach can be used to good effect to target multiple brain sites with considerable precision, and the results extend our understanding of effective connectivity in the primate brain.

In conclusion, our observations provide evidence for different forms of effective connectivity within the auditory ventral processing stream. We obtained evidence that stimulation of an anterior voice-sensitive region in the aSTP does not elicit significant functional activity in the frontal cortex but appears to engage primarily an anterior ipsilateral temporal lobe network. These results are in stark contrast to those obtained by stimulating upstream auditory areas in the lateral belt or a presumed further downstream site in the multisensory aSTS, all of which result in activation of frontal cortex. The findings suggest that certain brain regions in the primate ventral temporal pathway rely on adjacent processes before gaining access to the frontal cortex, at least in the anesthetized preparation. The results combine with other notable exceptions, primarily obtained in the visual system, challenging a unique form of organization of the different processing streams in the brain.

## Methods

### Study subjects

Four adult male rhesus monkeys (*Macaca mulatta*) were studied, age ranged from 5 to 7 years. All procedures were approved by the local authorities (Regierungspräsidium Tübingen, Germany; Referat 35, Veterinärwesen) and were in full compliance with the guidelines of the European Community (EUVD 86/609/EEC) for the care and use of laboratory animals. The sample size was chosen to minimize the numbers of animals studied while ensuring that the key observations are supported in at least two–three of the animals.

### Anaesthesia protocol

An extensive description of the handling and anaesthesia procedures was reported previously^16^,^30^,^31^,^37^,^38^,^39^ (also see Supplementary Text). In brief, the handling and anaesthesia protocols ensure stress-free treatment of the animals, while, at the same time, preserving neural responses to sensory stimulation. The animals were premedicated with glycopyrrolate (intramuscular 0.01 mg kg^−1^) and ketamine (intramuscular 15 mg kg^−1^), and then a catheter was inserted into the saphenous vein. Animals were then preoxygenated and prepared for intubation with a combination of short-acting drugs (fentanyl at 3 μg kg^−1^, thiopental at 5 mg kg^−1^ and the muscle relaxant succinyl-choline chloride at 3 mg kg^−1^). The trachea was then intubated and the lungs were ventilated at 25 strokes per min. We maintained anaesthesia with remifentanil (0.5–2 μg kg^−1^ min^−1^) in combination with a fast acting paralytic, mivacurium chloride (5 mg kg^−1^ h^−1^). Because the fMRI BOLD signal is very sensitive to changes in body temperature, oxygenation, pH and blood pressure, the physiological state of the animal was monitored continuously and maintained tightly within the normal limits. Body temperature was strictly maintained at 38–39 °C, and end-tidal CO_2_ and oxygen saturation were kept constant at 33 mm Hg and over 95%, respectively. Acidosis was prevented by administering lactated Ringer’s solution with 2.5% glucose, infused at 10 ml kg^−1 ^h^−1^. Intravascular volume was maintained by administering colloids (hydroxyethyl starch, 20–30 ml over 1–2 min or 20 ml kg^−1^h ^−1^). We have measured catecholamines and optimized dosages to ensure unaffected physiological responses at normal catecholamine concentrations^38^. Functional data acquisition started ~2 h after the start of animal preparation, following acquisition of a high-resolution anatomical scan. The Supplementary Text has a more extensive consideration of the effects of the anaesthesia protocol on the BOLD response.

### MRI and fMRI data acquisition

Measurements of the fMRI blood oxygen level dependant (BOLD) signal were made on a non-human primate dedicated, vertical 4.7-Tesla MRI scanner (Bruker BioSpin, Ettlingen, Germany). Signals were acquired using a birdcage radiofrequency coil. The animals were scanned while seated in a customized primate scanning chair with their head held by a stereotactic device. Functional MRI data were acquired using a gradient-recalled echo planar imaging sequence with the following typical parameters: echo time, TE: 20 ms; TR: 1.5 s; flip angle: 60°; 22 slices, 2-mm thickness; in-plane field of view: 9.6 × 9.6 cm^2^, on a grid of 128 × 128 voxels, with a voxel resolution of 0.75 × 0.75 × 2 mm^3^. Anatomical images for localizing the electrode were obtained in axial, coronal or sagittal planes using T2-weighted FLASH (fast low-angle shot) sequences (Supplementary Fig. S1) with typical parameters TE: 10 ms; TR: 1,000 ms; flip angle: 60°; 22–35 slices; in-plane field of view: 9.6 × 9.6 cm^2^, on a grid of 256 × 256 voxels. Anatomical scans in register with each functional scanning experiment were also obtained using a three-dimensional T1-weighted MDEFT (modified driven equilibrium Fourier transform) sequence with typical parameters TE: 5 ms; TR: 20 ms; flip angle: 20°; 128 slices; in-plane field of view: 12.8 × 12.8 cm^2^, on a grid of 256 × 256 voxels, with typical voxel resolution of 0.5 × 0.5 × 0.5 mm^3^; four segments.

### Functional MRI localizers

Before the microstimulation experiments, the four animals (M1–M4) underwent fMRI localizer experiments, as follows. The functional localization experiments included tonotopic auditory cortex mapping using tones and band-passed noise at different center frequencies^37^,^56^. Analyses identifying the reversals of tonotopic sound-frequency preference gradients and the approximate location of the core auditory cortex were used to delineate auditory cortical fields (ACF; Supplementary Fig. S5). Briefly, sounds at different frequencies elicit frequency selective fMRI activity patterns throughout the auditory core and belt. Gradient analyses of the tonotopic gradient reversals reveal the approximate location of borders between core and belt fields in the anteroposterior direction. A primary auditory cortex localizer based either on comparing tone versus band-passed noise responses (the latter of which are stronger in the auditory belt) and/or using thresholded tone responses (which are stronger in the core) helps to distinguish auditory core versus belt fields. Auditory parabelt or downstream fields are not thought to be tonotopically organized and are delineated in relation to the position between the tonotopically organized core and belt fields and the remaining anatomical regions thought to reside on the STP. For additional details please see refs 16, 37, 56.

**Figure 1:**
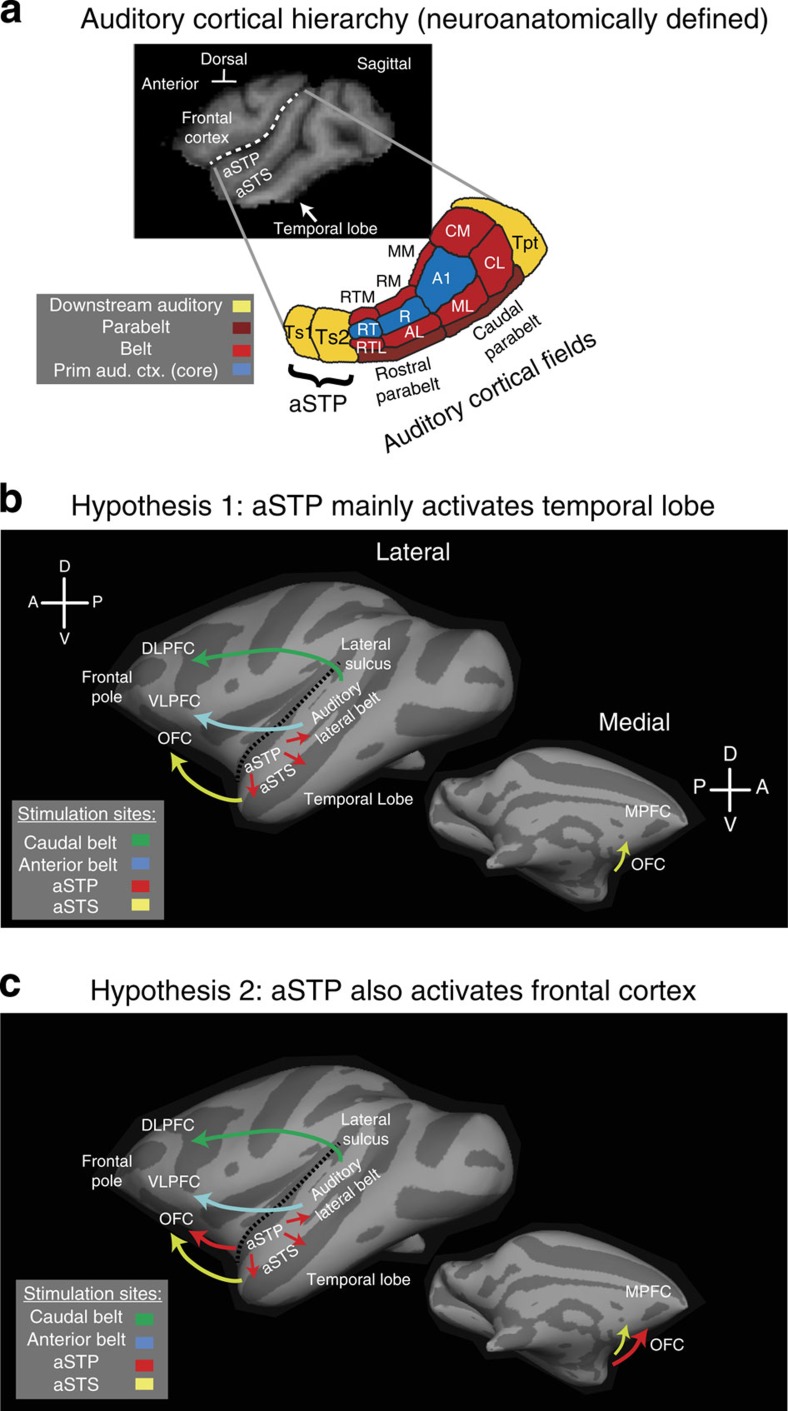
Auditory cortical processing stages and illustrated hypotheses. (**a**) Schematic of the known macaque auditory cortical hierarchy. For the definition of abbreviations for the different auditory cortical fields, see ref. 49. More ventral to these auditory regions (in the upper bank of the superior temporal sulcus, STS) are multisensory regions and further ventral (in the fundus and lower bank of the STS) are visual areas. (**b**,**c**) Illustrated hypotheses of aSTP effective connectivity. (**b**) Hypothesis 1 illustrates that stimulation of aSTP (red arrows) results mainly in anterior temporal lobe activity. (**c**) Hypothesis 2 illustrates that aSTP stimulation (red arrows) also activates orbital/medial frontal cortex (OFC/MPFC). Note that stimulation of other brain areas such as the lateral belt can help to evaluate to what extent combined microstimulation and fMRI recapitulates key findings from neuronal tractography studies. Thereby, in both hypotheses, we illustrate findings that could be consistent with neuronal tractography results, such as: ventrolateral prefrontal cortex (VLPFC) projections from anterior lateral belt fields^5^,^25^ in blue arrows and dorsolateral prefrontal cortex (DLPFC) projections from caudal lateral belt fields^5^,^25^ in green arrows; also shown in yellow arrows are aSTS projections to OFC or medial prefrontal cortex (MPFC)^8^,^27^; see text for further details.

We also mapped anterior voice-sensitive clusters using voice versus non-voice and, time permitting, voice-identity adaptation localizers^16^. In brief, the fMRI activity response to a stimulus category of macaque vocalizations produced by many individuals (that is, many voices) is compared with a stimulus category of non-voice sounds (that is, natural and environmental sounds; see Supplementary Figs 1–4). Since our hypotheses were specifically for voice-sensitive clusters in the Ts1/Ts2 regions anterior to the tonotopically organized core and belt fields (Fig. 1), we restricted our analysis of voice-sensitive clusters to the Ts1/Ts2 region (Supplementary Figs 1–4). Thus, the strongest voice-sensitive clusters in these regions were targeted for microstimulation. For additional details on the fMRI voice localization procedure please see refs 16, 17. Finally, in one experiment with macaque 3 we were also able to identify an anterior face-sensitive cluster in the fundus of the STS using a face versus non-face fMRI localizer^38^,^57^ (Supplementary Fig. 10).

### Microstimulation approach

**Figure 2:**
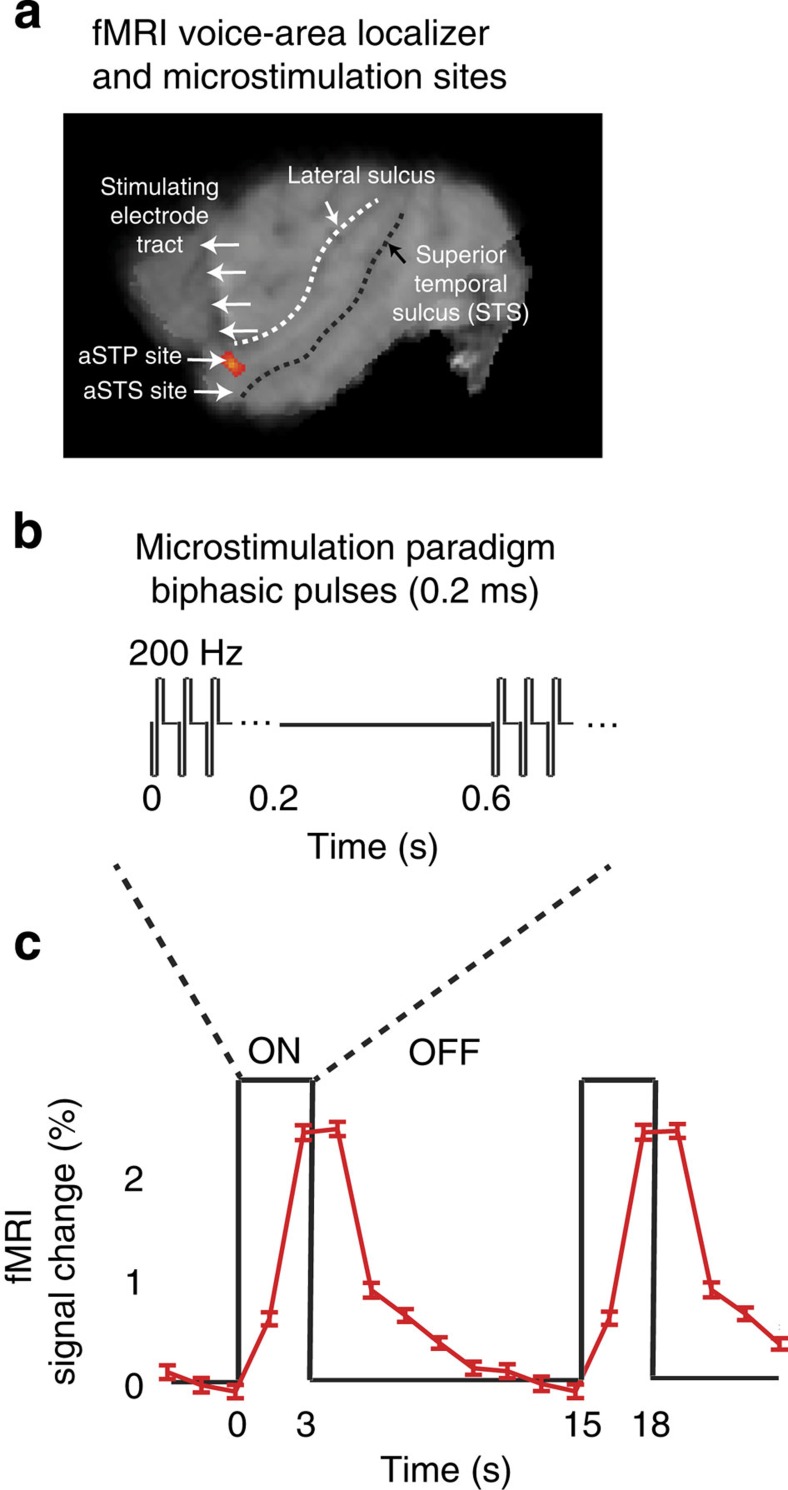
Anterior superior temporal regions targeted for microstimulation and the approach. (**a**) illustrates the left hemisphere approach (in monkey 1, M1) for targeting either the anterior voice-sensitive cluster, localized to the aSTP (in fields Ts1/Ts2 on the plane), or a more ventral site in the upper bank of the aSTS (in field TPO^24^). The aSTP voice-sensitive cluster (in red) is based on a separately obtained voice versus non-voice fMRI localizer (see Supplementary Figs 1–4 for additional examples). The position of the electrode can be identified by its local signal dropout (see text for further details). (**b**) Schematic of the microstimulation paradigm showing periods of biphasic stimulation alternating with no-stimulation periods. (**c**) Illustrative time course of the fMRI BOLD signal in the aSTS in response to stimulation of the aSTP (from the experiment shown in Fig. 3a).

**Figure 3:**
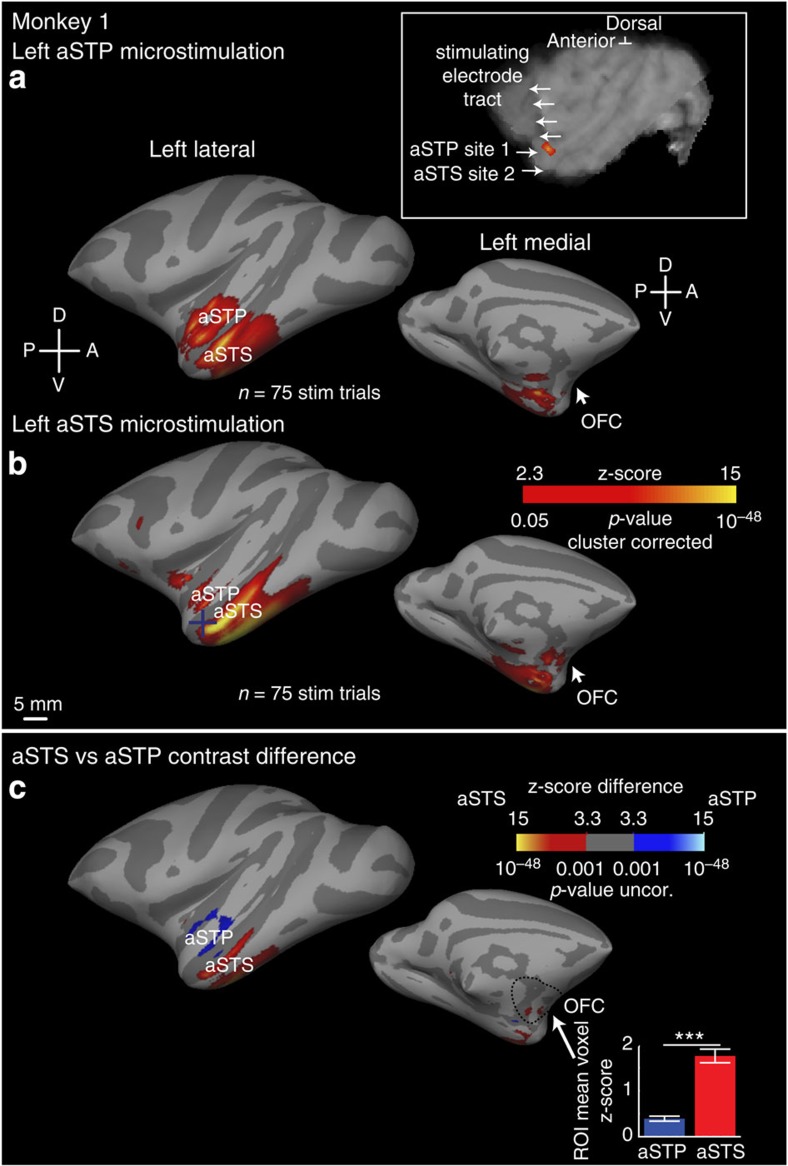
Comparison of the effects of aSTP and aSTS stimulation in Monkey 1. (**a**) Significantly activated clusters (cluster corrected *P<*0.05) in Macaque 1 (M1) resulting from stimulating the voice-sensitive cluster in the aSTP (Supplementary Fig. 1; crosshairs identify site of stimulation). Results are shown on a surface-rendered macaque template brain (gyri, light grey; sulci, dark grey). (**b**) Significantly activated clusters resulting from aSTS microstimulation. (**c**) Analytical contrast between the effects of stimulating the aSTS versus aSTP sites. Bar graphs show the results of anatomically defined ROI analyses (shown is the ROI mean voxel *z* score, ±s.e.m., across trials). The results show that the OFC ROI is consistently more activated by aSTS than by aSTP stimulation (Supplementary Table 1 summarizes the anatomical regions activated). No significantly activated voxels were observed in the contralateral (right) hemisphere.

**Figure 4:**
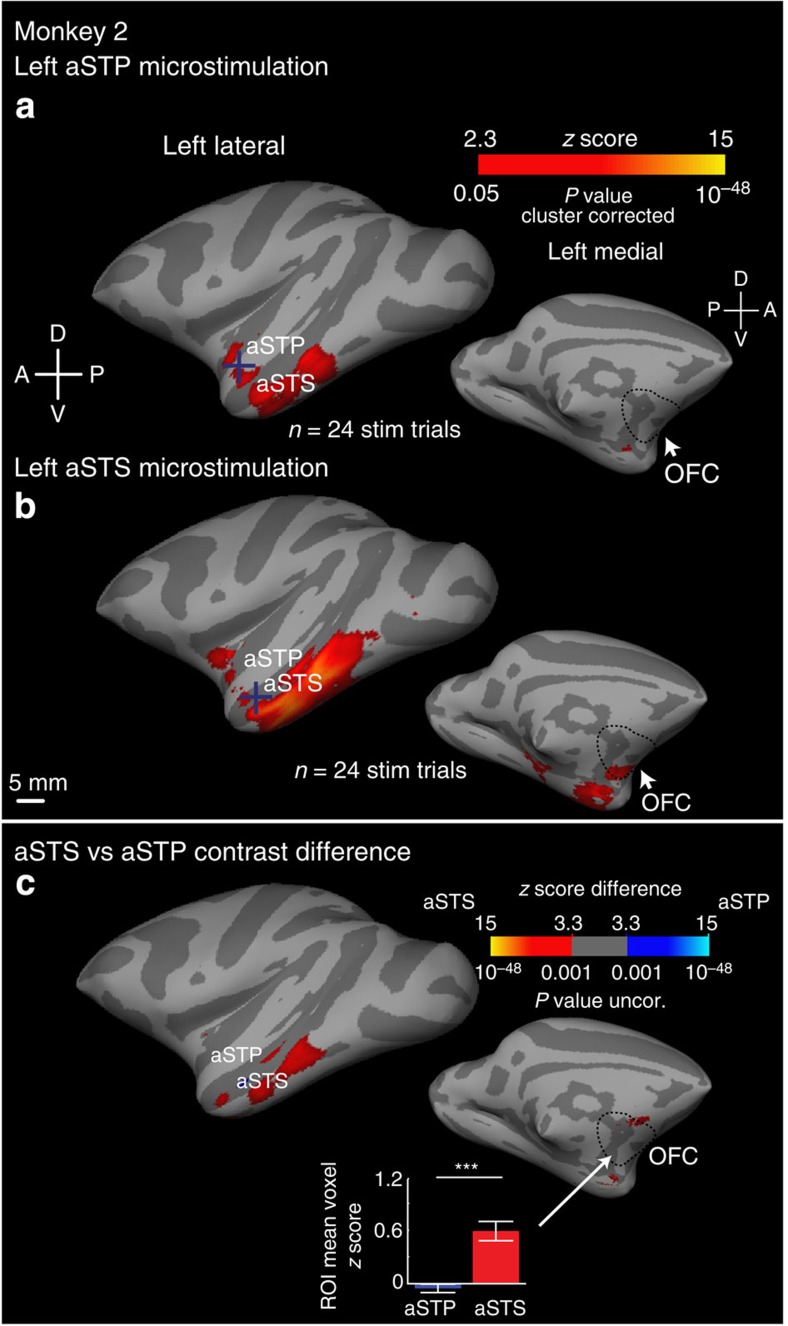
Comparison of the effects of aSTP and aSTS stimulation in Monkey 2. Shown are the significantly activated clusters (cluster corrected *P<*0.05) in Macaque 2 (M2) resulting from stimulating the voice-sensitive aSTP (**a**) or the aSTS (**b**). (**c**) Analytical contrast between the effects of stimulating the aSTS versus aSTP sites. Format as in Fig. 3. No significantly activated voxels were observed in the contralateral (right) hemisphere. See manuscript Supplementary Table 2 for a summary of the significantly activated anatomical regions.

**Figure 5:**
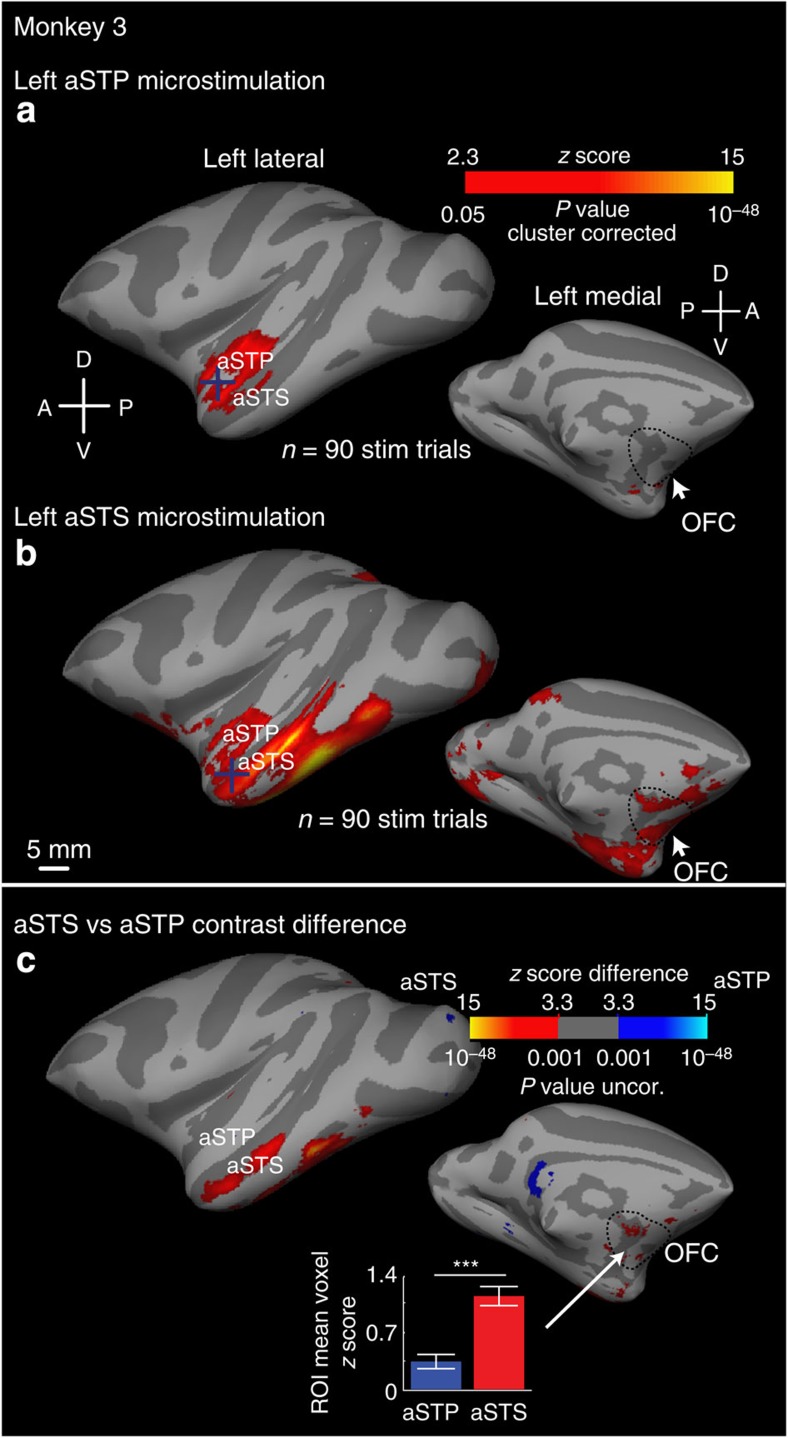
Comparison of the effects of aSTP and aSTS stimulation in Monkey 3. Shown are the significantly activated clusters (cluster corrected *P<*0.05) in Macaque 3 (M3) resulting from stimulating the voice-sensitive aSTP (**a**) or the aSTS (**b**). (**c**) Analytical contrast between the effects of stimulating the aSTS versus aSTP sites. Format as in Fig. 3. No significantly activated voxels were observed in the contralateral (right) hemisphere. See manuscript Supplementary Table 3 for a summary of the significantly activated anatomical regions.

**Figure 6:**
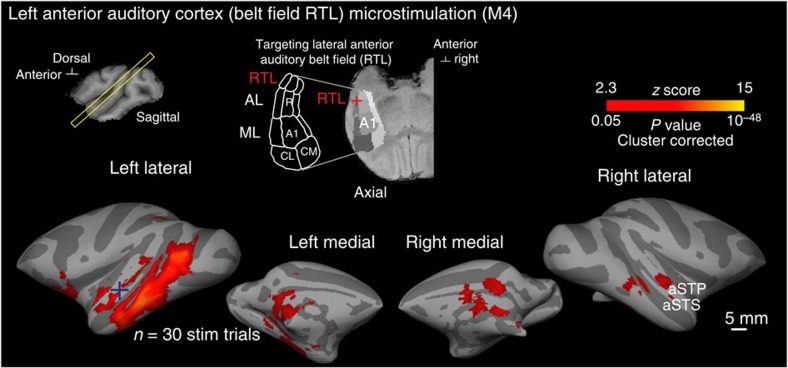
fMRI results from stimulating an anterior lateral belt field. Top left panels show the tonotopically organized auditory core and belt fields, which were localized separately using fMRI activity in response to tones and band-passed noise varying in frequency^37^. Bottom panels show the result of microstimulating the anterior lateral belt field RTL. Note the prominent cross-hemisphere activation and significant activation of orbitofrontal and ventrolateral frontal cortex. Supplementary Table 4 summarizes the anatomically activated regions resulting from stimulation of this region in the lateral belt.

**Figure 7:**
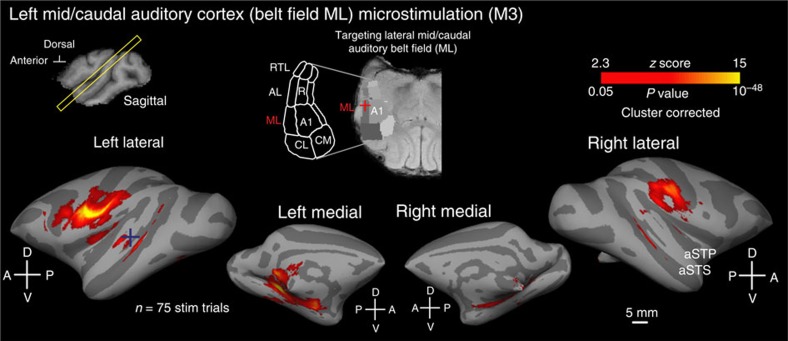
fMRI results from stimulating a mid/caudal lateral belt field. Format as in Fig. 6. Top left panels show the tonotopically organized auditory core and belt fields^37^. Bottom panels show the result of microstimulating the mid/caudal lateral belt field ML. Note the prominent cross-hemisphere activation and significant activation of ventral and dorsolateral frontal cortex. Supplementary Table 5 summarizes the anatomically activated regions resulting from stimulating this region in the lateral belt.

**Figure 8:**
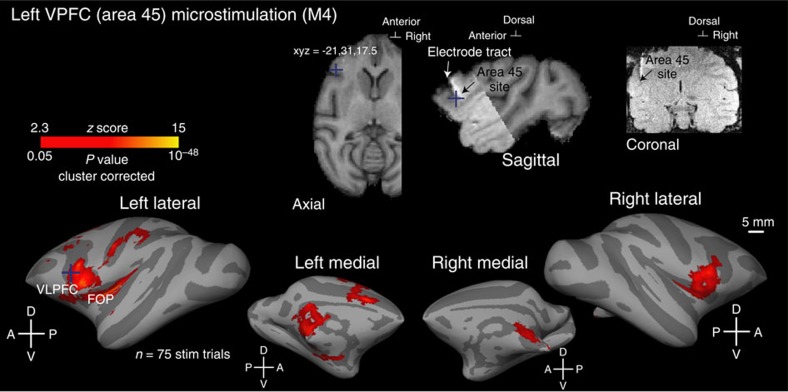
fMRI results from microstimulating frontal cortex area 45. Format as in Fig. 6. Top right panels show the electrode position and targeting approach for this experiment. Bottom panels show the results from stimulating area 45. See manuscript text for details and Supplementary Table 6 for a summary of the significantly activated anatomical regions. FOP, frontal operculum; VLPFC, ventrolateral prefrontal cortex.

**Figure 9:**
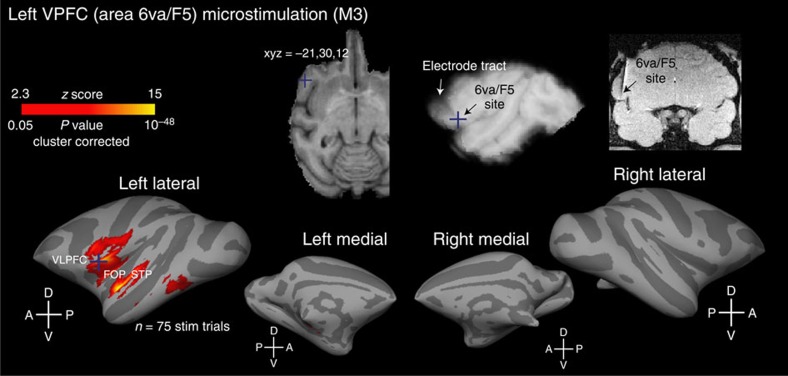
fMRI results from microstimulating frontal cortex area 6va/F5. Format as in Fig. 8. Top right panels show the electrode position and targeting approach for this experiment. Bottom panels show the results from stimulating area 6va/F5, which is more ventral than the area 45 site shown in Fig. 8. See manuscript text for details and Supplementary Table 7 for a summary of the significantly activated anatomical regions.

**Figure 10:**
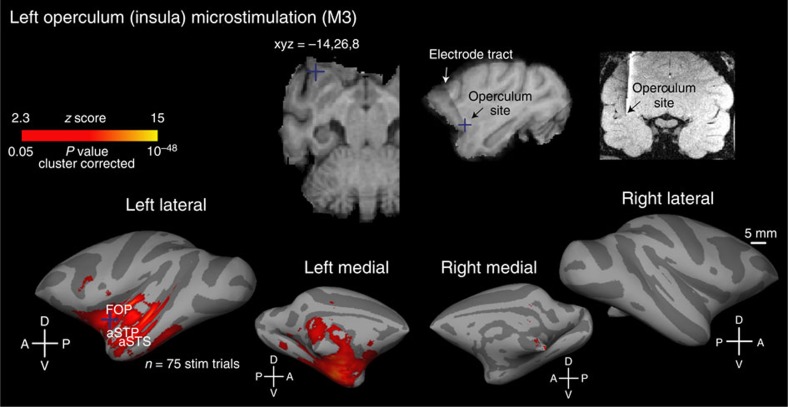
fMRI results from microstimulating the frontal operculum. Format as in Fig. 8. Top right panels show the electrode position and targeting approach for this experiment. Bottom panels show the results from stimulating the frontal operculum (FOP, compare with Figs 8 and 9). See manuscript text for details and Supplementary Table 8 for a summary of the significantly activated anatomical regions.

Chambers to target fMRI-identified clusters or other stereotactically determined anatomical sites were implanted using neurosurgical targeting approaches^17^. We targeted for microstimulation the anterior voice-sensitive clusters in the aSTP, within anatomical areas Ts1/Ts2 (Fig. 2, Supplementary Figs 1–4). We also targeted an upper-bank aSTS anatomical region, which we observed was activated by aSTP stimulation (localized to anatomical field TPO; Figs 3, 4, 5; Supplementary Figs 1–4). The aSTS site was located 4–6 mm below the aSTP site and separated from it by white matter. As reported, for some experiments we also targeted sites within other anatomical areas, using structural MRI scans and stereotactic coordinates^24^.

Electrodes were custom-made platinum/iridium glass-coated electrodes (see below). MRI was used to identify the electrode position by the local signal loss caused by the platinum/iridium microfilament in the electrode (Fig. 2; Supplementary Figs 1–4). Because the tip of the electrode cannot be localized precisely, we stopped the approach to the target site 4–5 mm short of it and then slowly advanced the electrode while continually monitoring neuronal responses. This allowed us to evaluate the silent transition areas that the electrode passed through, such as those in the lateral sulcus and in the white matter between the aSTP and aSTS sites. Once neuronal activity increased again as the electrode entered the cortical grey matter, we advanced the electrode ~1 mm further so as to be well within the grey matter. An additional set of MRI scans was then acquired to identify the approximate electrode placement, although we could not identify its laminar location (Fig. 2; Supplementary Figs 1–4).

### Microstimulation procedure

The microstimulation procedure has been reported elsewhere in detail^30^,^31^,^53^ as have the methods we used for conducting simultaneous electrophysiological recordings and MRI^58^. In brief, induction voltages caused by gradient coil switching were compensated for by measuring the induced currents on the animal with rotationally symmetric sensors placed around the electrode. Current was passed back to the animal via a wire in the mouth to cancel the currents measured by the sensor (see ref. 58, for details). In addition, for this study we used a custom-built constant current source, with the aim of compensating for the capacitance of the cable that delivered the stimulation. The compensation circuit allowed calculating the instantaneous amount of current needed to charge the cable capacitance by measuring the differential of the voltage across the cable. The current needed to achieve this was then added to the desired current^30^,^31^,^53^.

We used glass-coated platinum-iridium electrodes, with impedances of 75–250 kΩ. Impedances were checked throughout the experiments and microstimulation was not conducted with electrodes that had impedances <75 kΩ. All reported experiments used a constant current of 500 μA. An experiment was terminated if a voltage threshold of 10 V was breached, which usually indicates that the electrode impedance had dropped below 75 kΩ and could compromise the quality of the results^31^. The current amplitude, pulse duration, train duration and stimulation frequency were controlled digitally by using the QNX real-time operating system (Fig. 2). We used a charge-balanced, biphasic-pulse procedure (consisting of square-wave pulse durations of 0.2 ms positive or negative)^30^,^31^. The stimulation frequency was 200 Hz, and included numerous non-stimulation periods to allow for neuronal refractory periods (Fig. 2). The stimulation protocol was presented in blocks, such that 10 (1.5 s) fMRI volumes were obtained during a stimulation ‘trial’, resulting in a trial length of 15 s. Each trial consisted of 4.5 s of no stimulation, 3 s of stimulation and 7.5 s of no stimulation (Fig. 2). The numbers of microstimulation trials obtained with fMRI are reported in the figures. Also, all analytical comparisons between the aSTP and aSTS experiments were matched in a numbers of trials.

### Functional MRI analyses

For each stimulation experiment, we performed a fixed-effects General Linear Analysis (FEAT, FSL^59^) contrasting fMRI BOLD responses to stimulation versus no-stimulation periods. The analysis of stimulation versus no-stimulation was evaluated using a hemodynamic response model and evaluated at the cluster corrected (*P<*0.05) level (2 mm smoothing full-width half maximum). The contrast between the effects of stimulating aSTS and aSTP (Fig. 2) is shown at the *P<*0.001 uncorrected level simply for consistency across all animals: we confirmed that the observation of aSTS versus aSTP stimulation resulting in greater orbitofrontal activity was also significant at the *P<*0.05 cluster-corrected level in the majority of animals and experiments (for M1 in Fig. 3 and M3 in Fig. 5 and for the data shown in Supplementary Fig. 6; also see Supplementary Tables 1–3 and 9–12 for voxels summary statistics).

With the animal’s own anatomical scans serving as intermediates, the results of these analyses were registered to a standard macaque template brain^60^, which is registered to a macaque atlas in stereotactic coordinates^24^ as well as a digital atlas developed from it^61^. This allowed us to determine the anatomical areas within which the significant activity clusters occurred. The results were also registered to a FreeSurfer (http://surfer.nmr.mgh.harvard.edu/) surface-based representation of the standard template monkey brain^60^. Anatomically defined ROIs in the anterior auditory cortex (rostral core/belt fields) or OFC were defined in reference to the macaque brain atlas^24^,^61^. For statistically testing the voxel-based responses in the ROIs, the ROIs were registered back to the animals’ own functional imaging data. This helped avoid overinflating the number of voxels used for analysis (which would happen if instead we had registered the functional scans to the higher resolution anatomical space and used this for the ROI analyses). These ROI data were also analysed with the animals’ data combined using a mixed-effects ANOVA with a random between-subjects factor of ‘monkey’ and a fixed within-subjects factor of 'stimulation site’. Hemisphere was also added to these models as needed (see Results for further details).

### Histological processing

At the end of all the experiments, each of the four animals was deeply anesthetized and trans-cardially perfused with saline followed by 4% paraformaldehyde in 0.1 M phosphate buffer (PB; pH 7.4).The formaldehyde-fixed brain was extracted, photographed and blocked in the coronal plane. The brain was stored in 10% glycerol with 2% dimethyl sulfoxide in 0.1 M PB and then transferred to 20% glycerol with 2% dimethyl sulfoxide in 0.1 M PB for up to 6 days. Frozen sections were cut in coronal planes at 40-μm thickness and adjacent sections were stained for Nissl (M1-4), SMI-32 or parvalbumin (M1). This allowed us to compare with the brain atlas histological slices^24^ and our MRI sections for further confirmation of some of the anatomical site positions (Supplementary Fig. 1).

## Additional information

**How to cite this article**: Petkov, C. I. *et al*. Different forms of effective connectivity in primate fronto-temporal pathways. *Nat. Commun.* 6:6000 doi: 10.1038/ncomms7000 (2015).

## Supplementary Material

Supplementary Information Supplementary Figures 1-10, Supplementary Tables 1-22, Supplementary Discussion, and Supplementary References
