# Supplementary material for: Different forms of effective connectivity in primate frontotemporal pathways
Source: Nat Commun. 2015 Jan 23;6:6000. doi: 10.1038/ncomms7000 (PMC4306228; doi:10.1038/ncomms7000)
Supplement: Supplementary Information — Supplementary Figures 1-10, Supplementary Tables 1-22, Supplementary Discussion, and Supplementary References [file ncomms7000-s1.pdf]

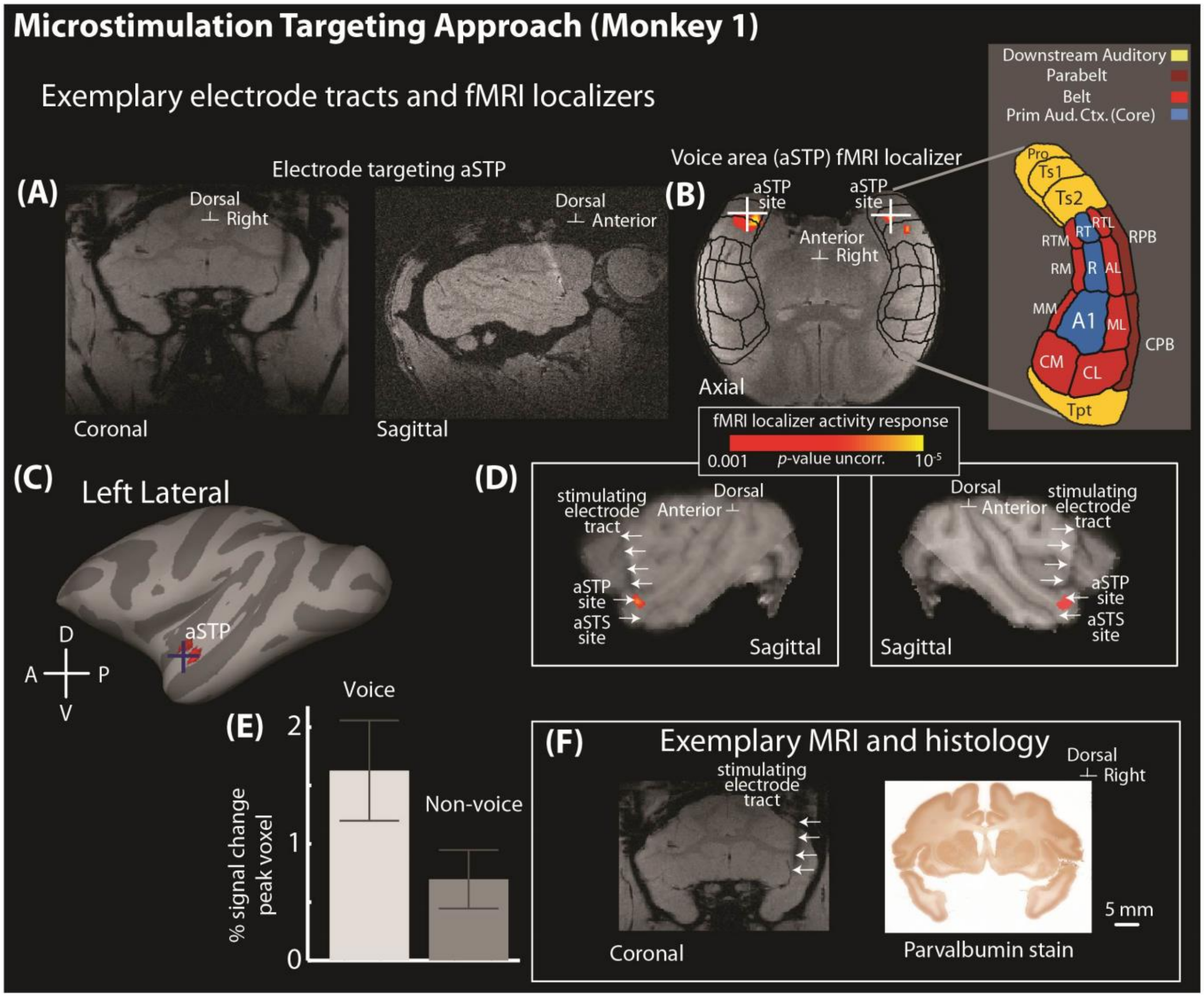

Supplementary Figure 1. Microstimulation site targeting approach in Macaque 1. (A-D) Illustrative results of fMRI localization identifying voice-sensitive clusters (red; masked with the approximate location of anatomical areas Ts1/Ts2, see B). Also shown is the targeting approach for the aSTP and aSTS sites using a combination of structural MRI images in axial, sagittal, and coronal planes. Neuronal activity was monitored in approaching each site to gain greater accuracy of electrode placement within the gray matter of the site of interest (see manuscript text). (E) plot of the % signal change relative to silent baseline (+/- Standard Error of the Mean, SEM, across trials, n = 172) of the response of the peak voxel in the left hemisphere cluster (see A-D) to the voice and non-voice localizer stimuli. (F) exemplary post-mortem parvalbumin stained coronal section, which was referenced with the MRI images of the monkey and the monkey atlas histology (see Fig. 74 in Saleem & Logothetis, 2007). This helped to further confirm that the microstimulated area was RTp, by the nomenclature of the atlas, or areas Ts2/Ts1 on the aSTP.

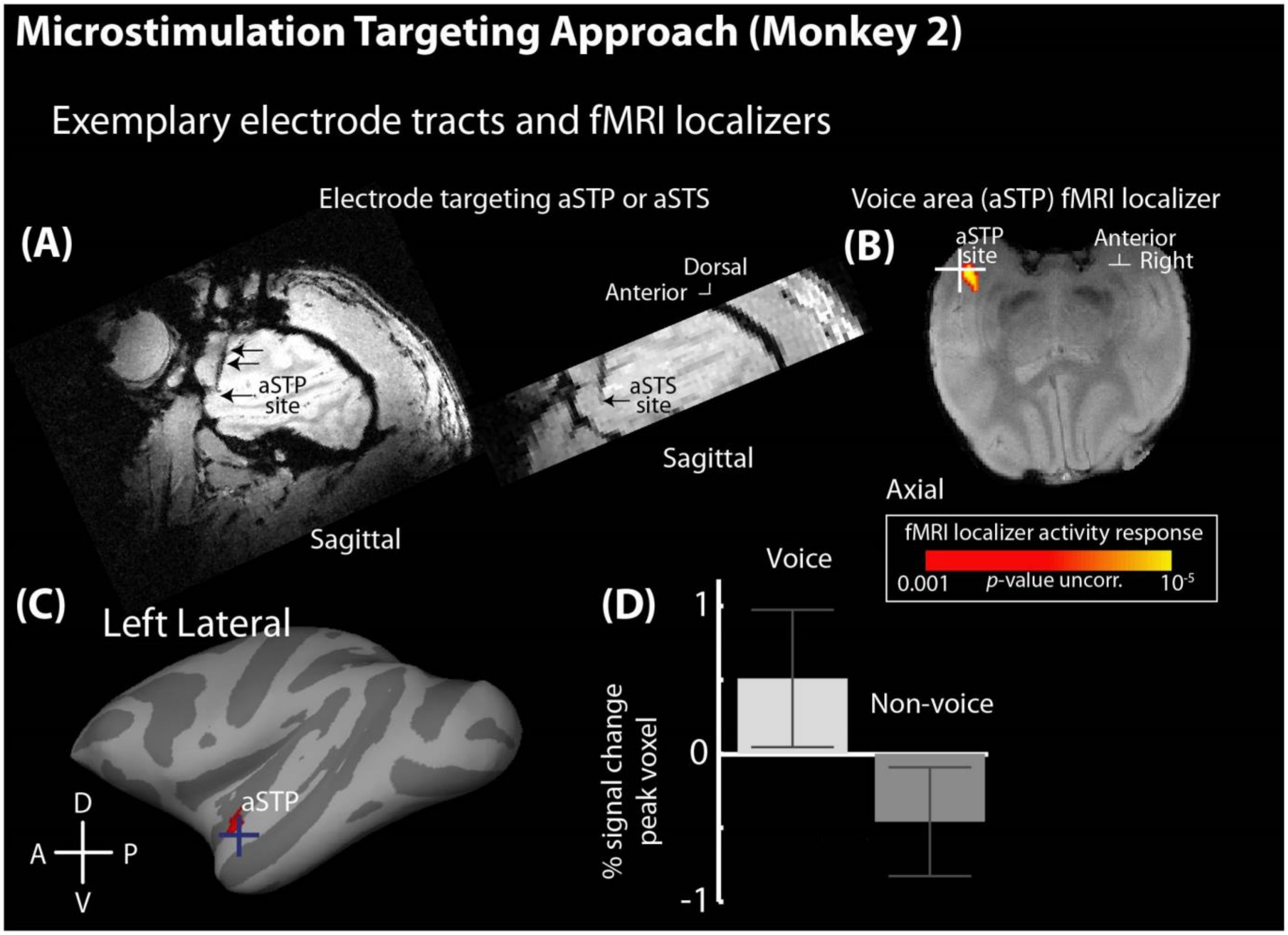

Supplementary Figure 2. Microstimulation site targeting approach in Macaque 2. (A-C) Illustrative results of fMRI localization identifying a voice-sensitive cluster and showing the targeting approach for the aSTP and aSTS sites. Format as in Suppl. Fig. 1. (D) plot of the % signal change relative to silent baseline (+/- SEM across trials, n = 136) of the response of the peak voxel in the left hemisphere cluster (A-C) to the voice and non-voice localizer stimuli.

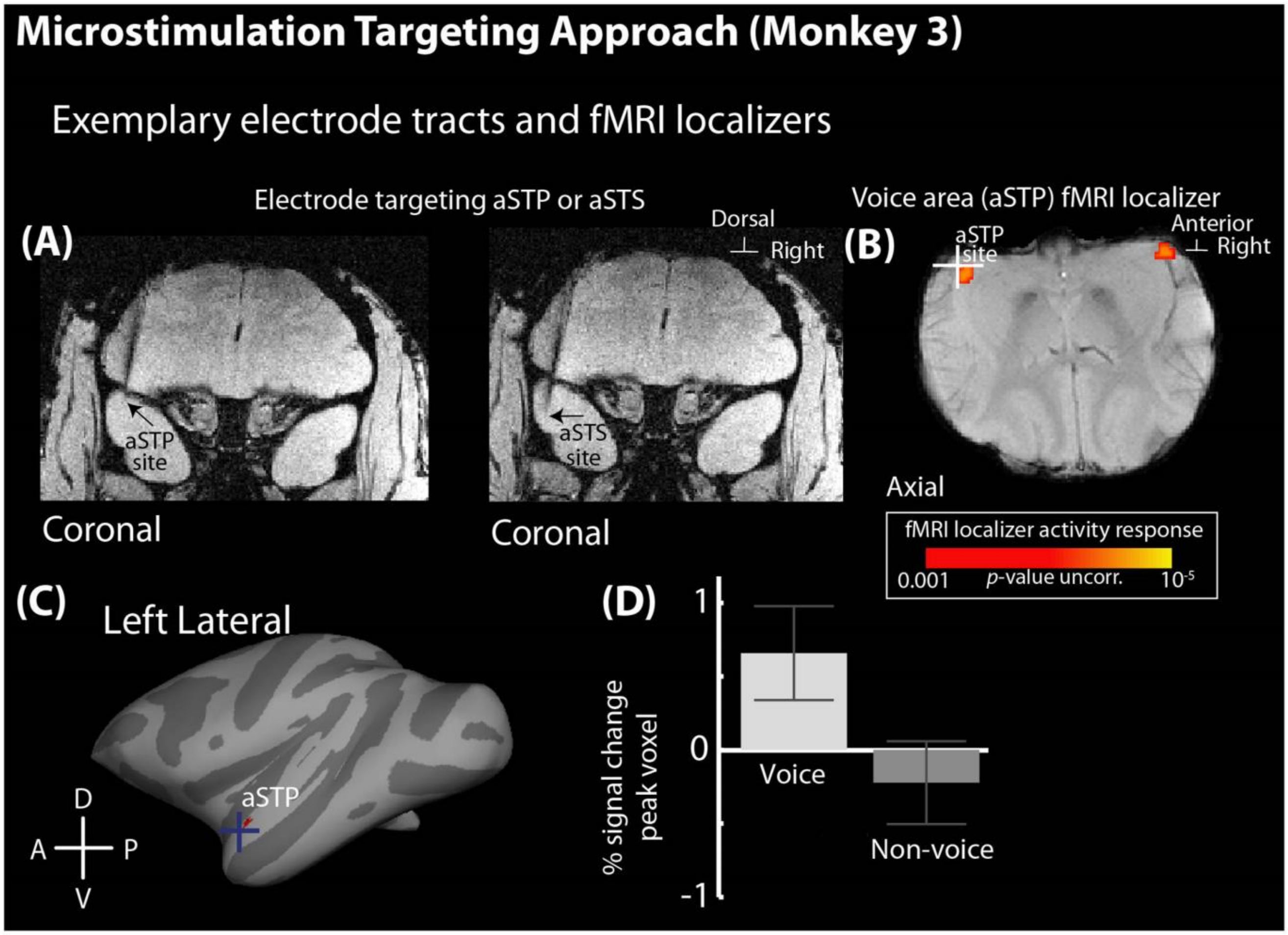

Supplementary Figure 3. Microstimulation site targeting approach in Macaque 3. (A-C) Illustrative results of fMRI localization identifying the voice-sensitive cluster and showing the targeting approach for the aSTP and aSTS sites. Format as in Suppl. Fig. 1. (D) plot of the % signal change relative to silent baseline (+/- SEM across trials, $n = 170$) of the response of the peak voxel in the left hemisphere cluster (A-C) to the voice and non-voice localizer stimuli.

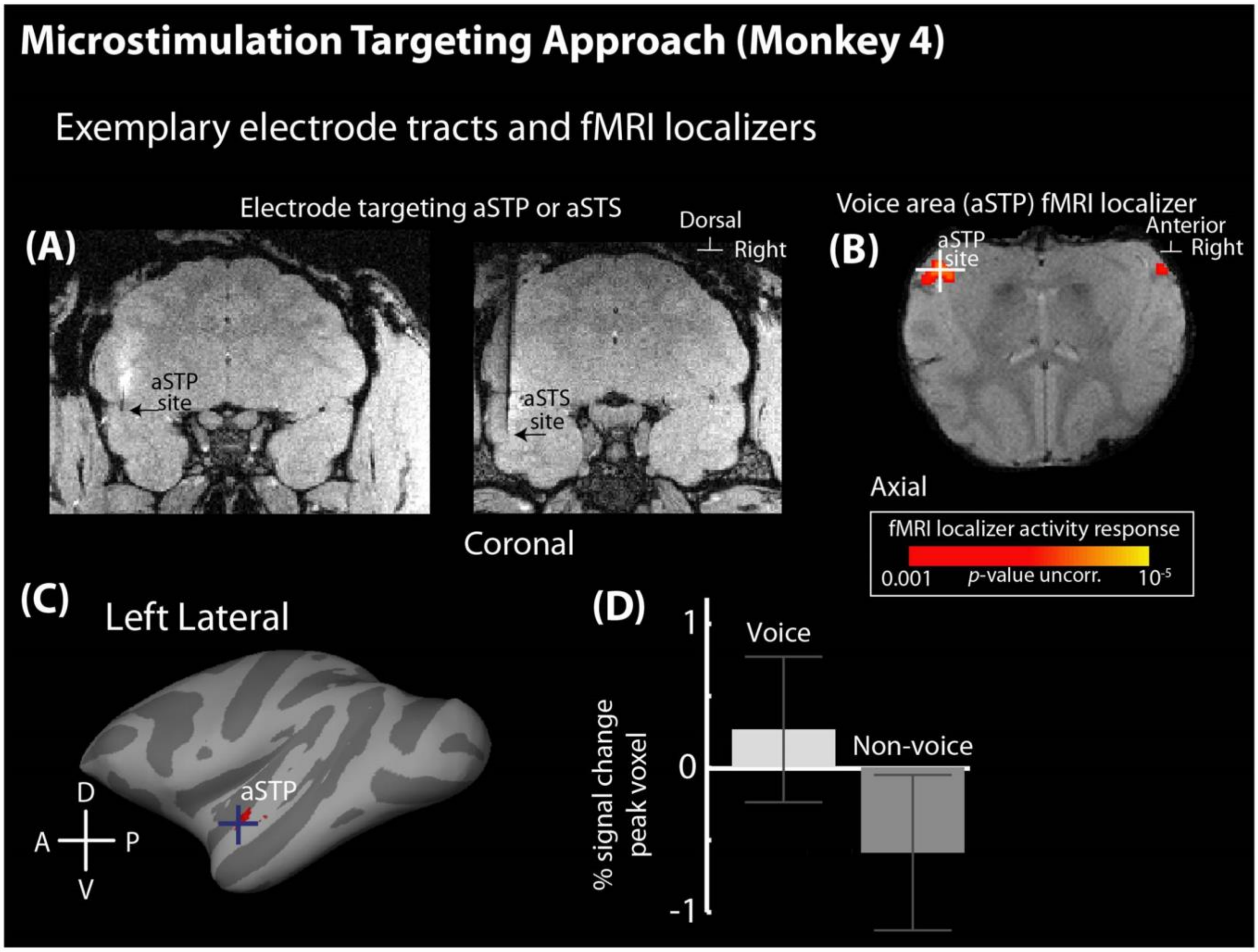

Supplementary Figure 4. Microstimulation site targeting approach in Macaque 4. (A-C) Illustrative results of fMRI localization identifying the voice-sensitive cluster and illustrating the targeting approach for the aSTP and aSTS sites. Format as in Suppl. Fig. 1. (D) plot of the % signal change relative to silent baseline (+/- SEM across trials, *n* = 170) of the response of the peak voxel in the left hemisphere cluster (A-C) to the voice and non-voice localizer stimuli.

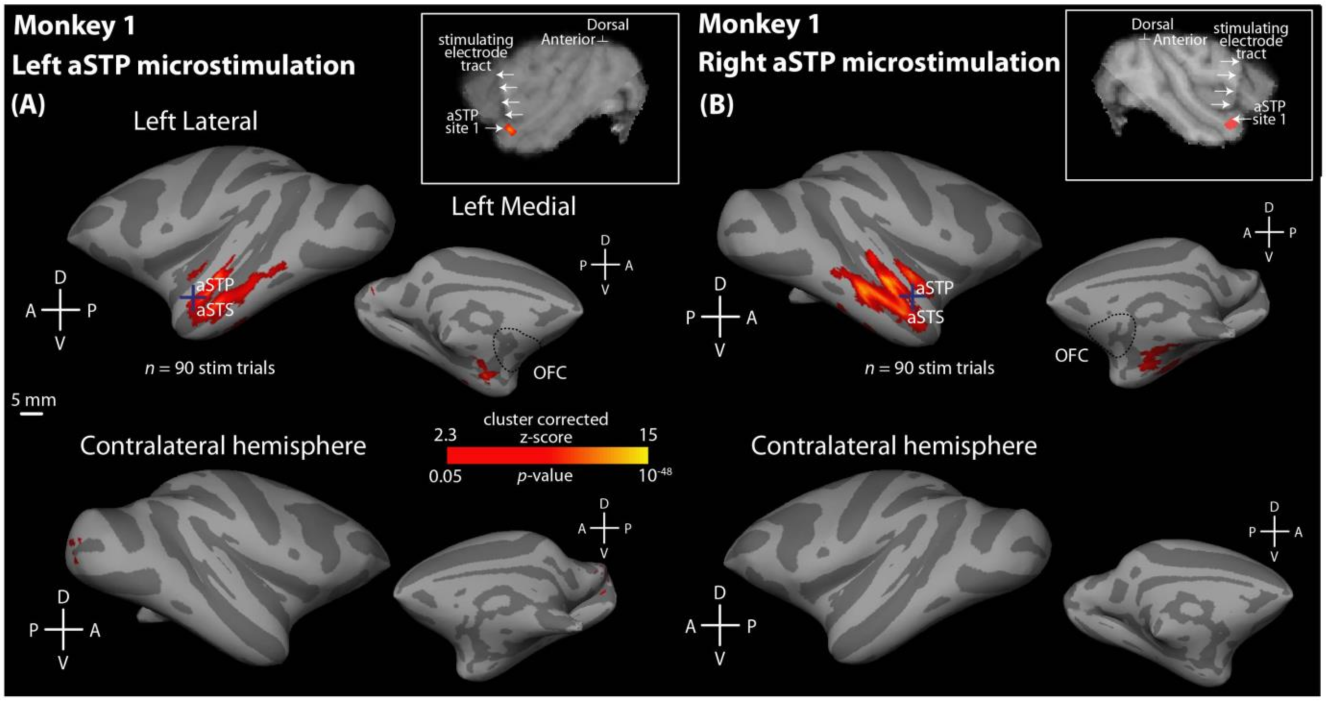

Supplementary Figure 5. Left and right hemisphere aSTP microstimulation results in M1. Format as in manuscript Fig. 3. This animal had both left and right chambers in order to target the aSTP in both hemispheres. Shown are the results of stimulating either the right or the left aSTP, which are comparable to those shown in manuscript Figs. 3-5 from M1-3.

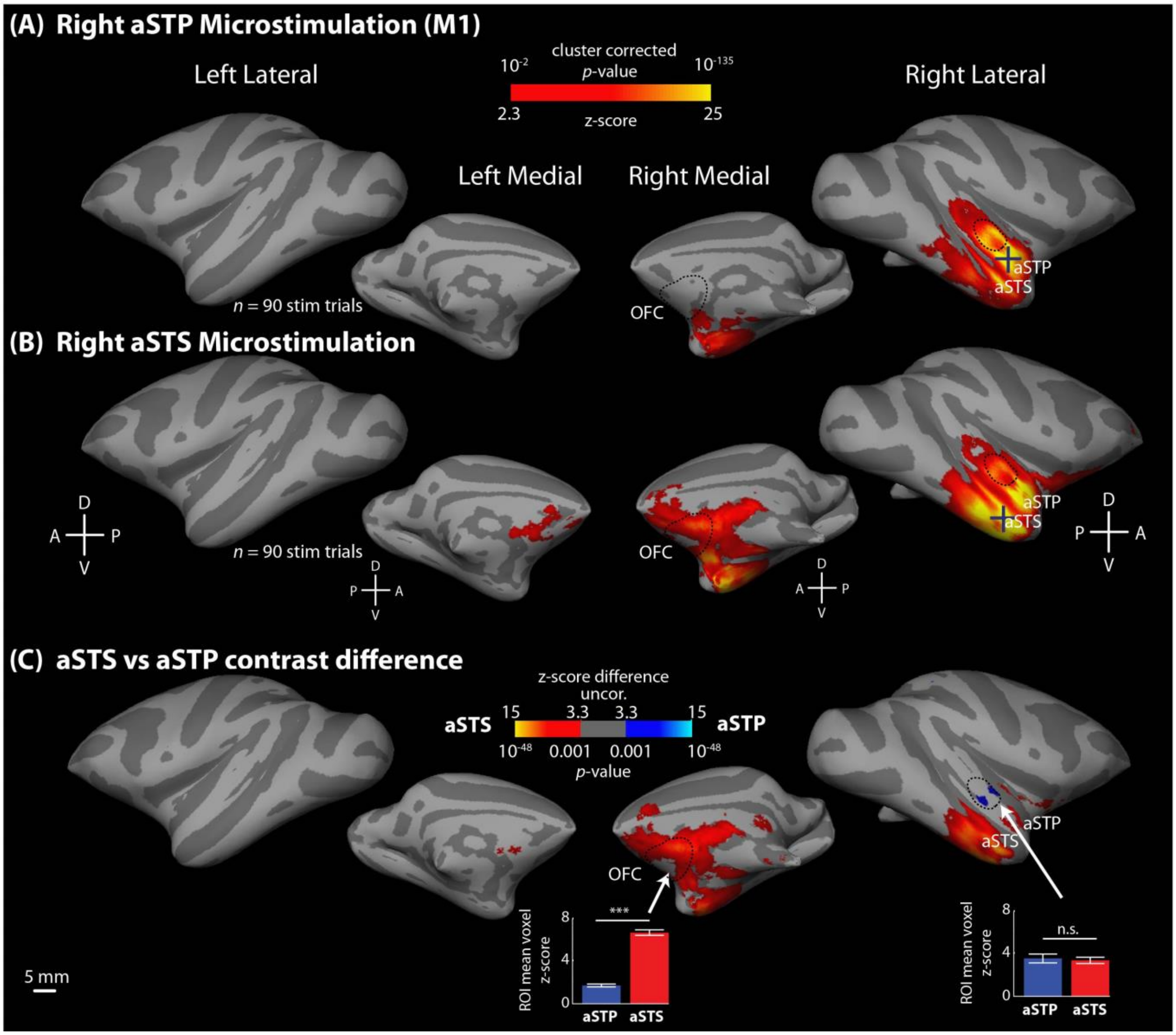

Supplementary Figure 6. Microstimulation of right hemisphere aSTP or aSTS in M1. Format as in manuscript Fig. 3. The effects of stimulating right hemisphere aSTP and aSTS sites replicate the effects of stimulating the left hemisphere in this animal (compare with manuscript Fig. 3). Namely, stronger OFC activation is seen by aSTS than by aSTP microstimulation. This was the only experiment of the 6 conducted to stimulate the aSTP (3 in manuscript Figs. 3-5 in M1-3; 2 in Suppl. Fig. 5 in M1 and this one in M1) where any frontal cortex activity other than in the frontal operculum was observed. Here, 11 significantly activated voxels were seen in the OFC by stimulating the aSTP; see Suppl. Table 11). However, by comparison, stimulation of the aSTS yielded hundreds of significantly activated voxels in frontal cortex, including many in the OFC (see Suppl. Table 11).

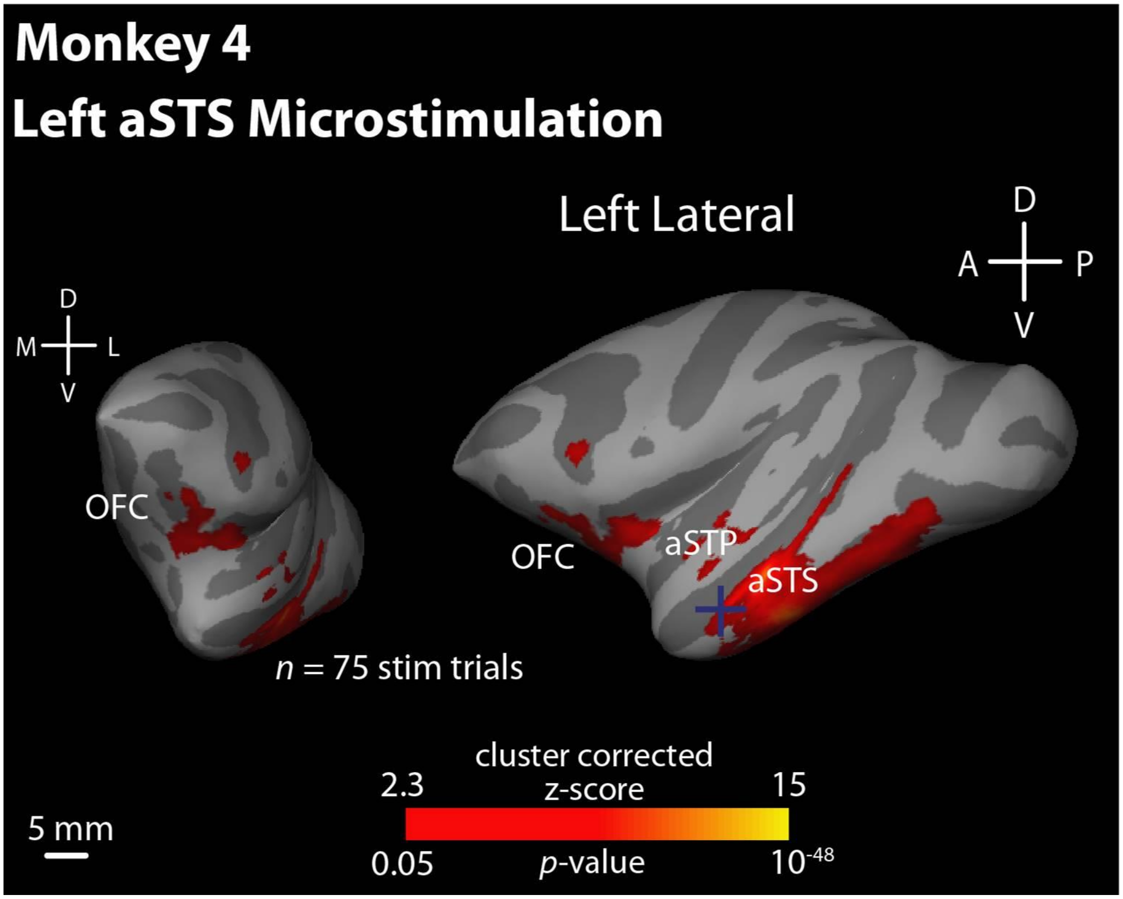

Supplementary Figure 7. Effects of microstimulating the aSTS in M4. The results from stimulating the *aSTP* in this monkey were considered a failure since they did not result in significant voxels anywhere in the brain, and they thus could not contribute to the aSTP vs aSTS comparisons in the manuscript. Nonetheless, the results from stimulating the *aSTS* in this animal resulted in significant activity, and the results correspond to those reported in the other 3 macaques where significant OFC activity was seen by microstimulating the aSTS (compare with manuscript Figs. 3-5; also see Suppl. Table 12).

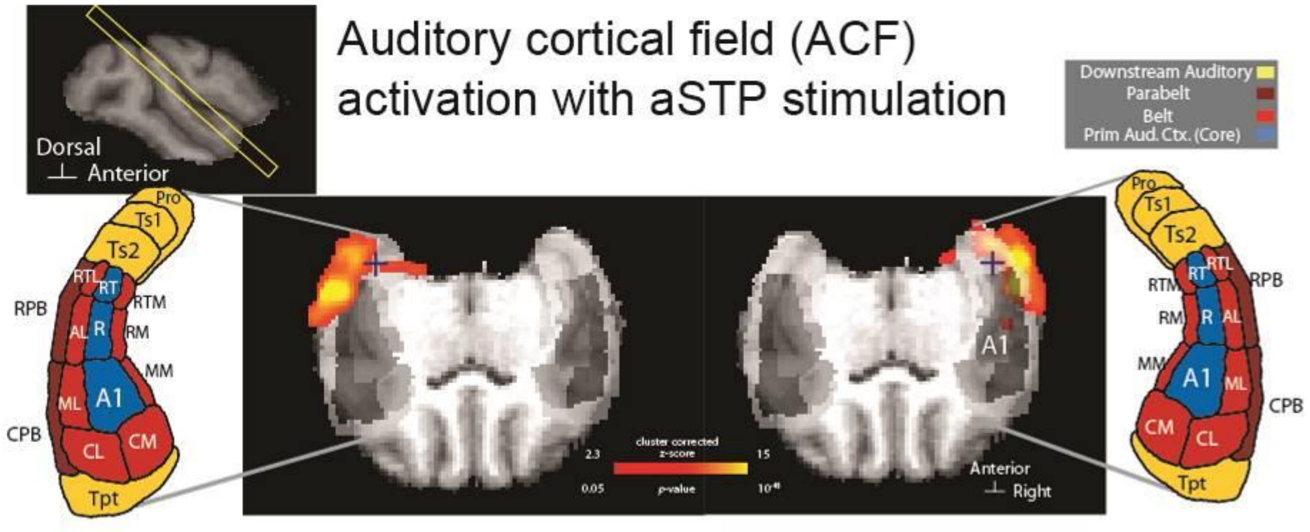

Supplementary Figure 8. Activated auditory cortical fields (ACFs) resulting from left and right hemisphere aSTP stimulation. Shows aSTP microstimulation results specific to activation of particular auditory cortical fields (ACFs) which are localized in a separate fMRI localizer experiment (see Methods). The results on the ACFs activated from stimulating the left or right aSTP in M1 were largely comparable with each other and resulted in activation of the more anterior ACFs of the ipsilateral hemisphere.

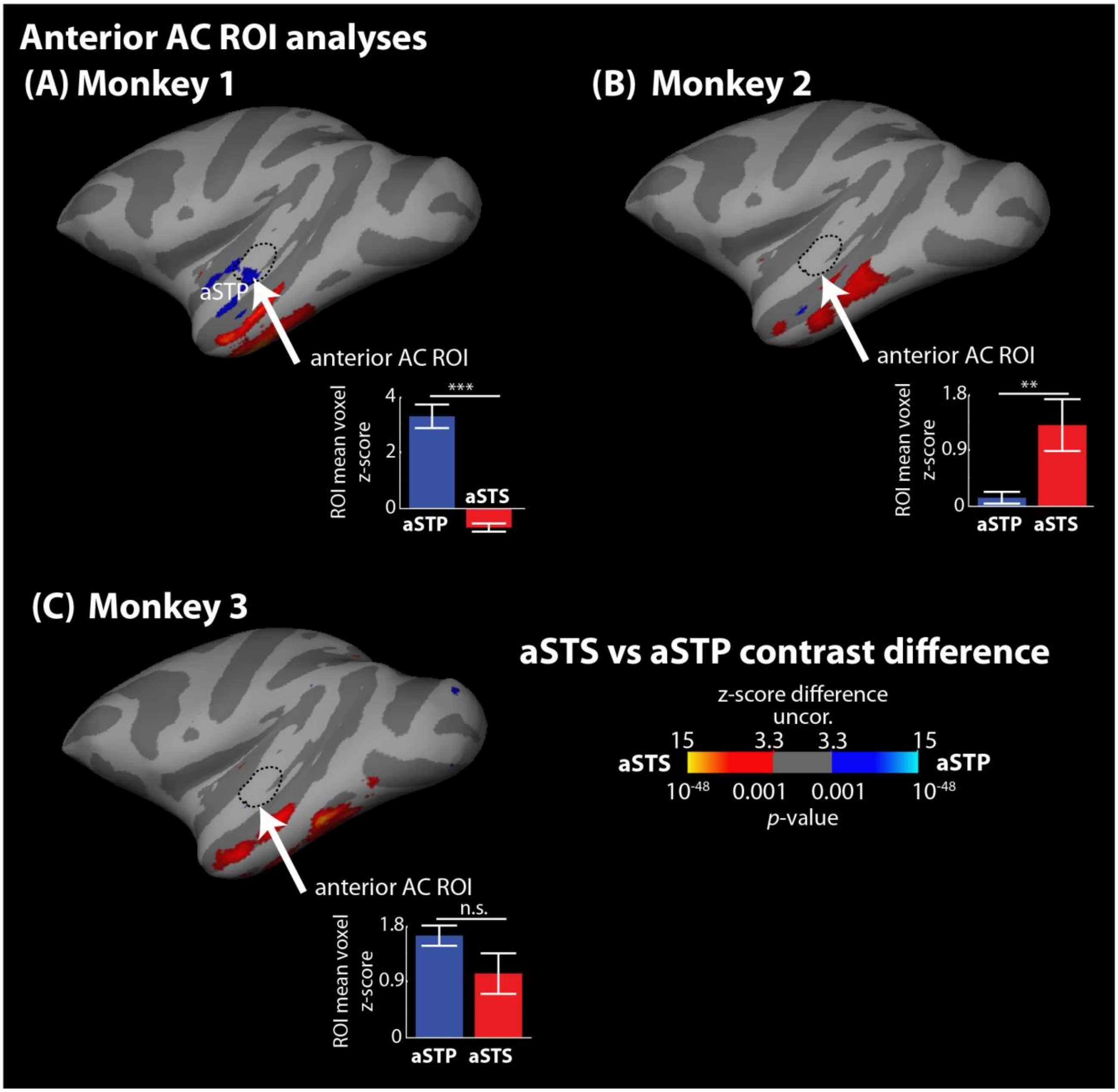

Supplementary Figure 9. Activation in an anterior auditory cortex ROI in response to aSTP or aSTS microstimulation. Format as in manuscript Fig. 3. In contrast to the consistently greater OFC activation across animals by aSTS than by aSTP stimulation, there was no consistent over- or under-activation of anterior auditory cortex (rostral core and belt fields; see Suppl. Fig. 8) by stimulating either the aSTP or aSTS.

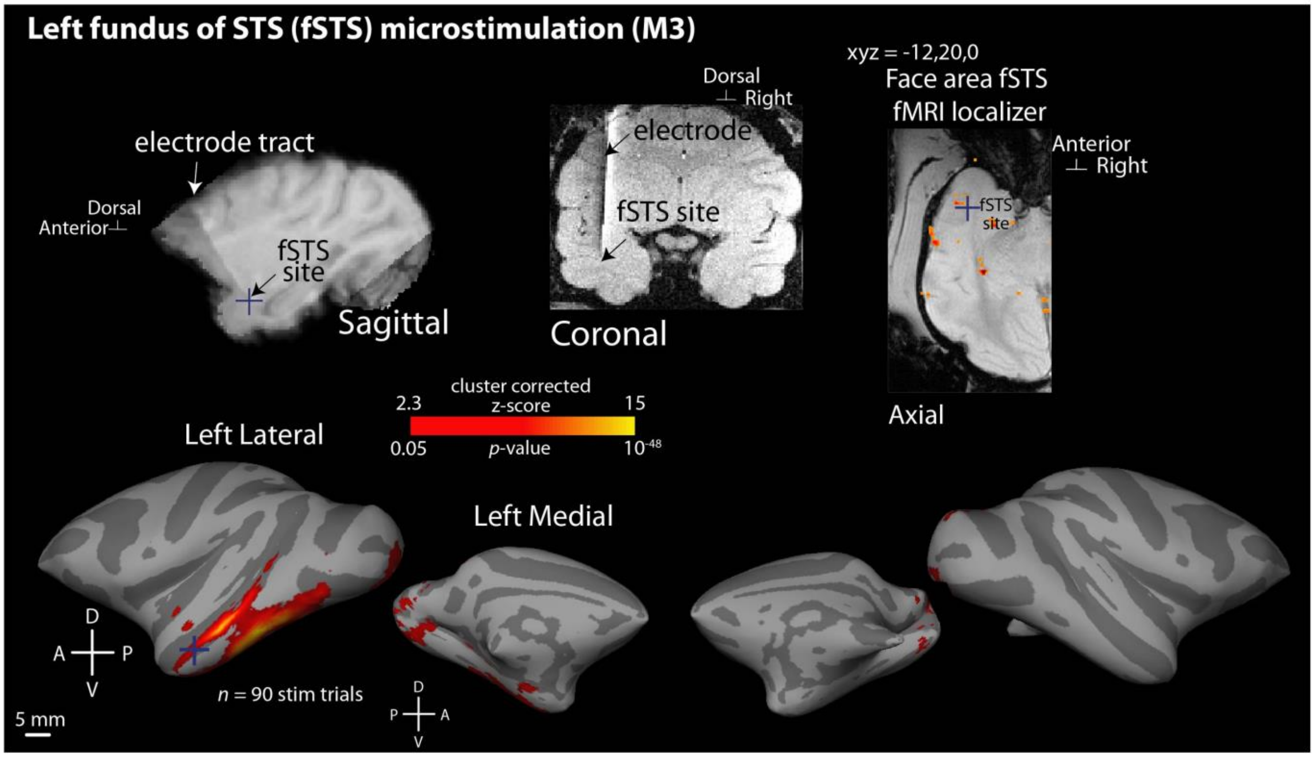

Supplementary Figure 10. Effects of stimulating a face-sensitive cluster in the fundus of the STS. Format as in manuscript Fig. 3. In one animal we localized an anterior face-sensitive cluster in the fundus of the STS, using a face vs. non-face fMRI localizer. We targeted this site for microstimulation, which resulted in widespread ipsilateral inferior temporal activation but no significant frontal cortex activity. Compare with results of a prior study of the inter-connectivity of macaque face-sensitive clusters using microstimulation and fMRI[1], which also did not show prominent activation of frontal cortex following any face cluster stimulation. Interestingly, that prior study provides an example of an upper-bank STS region that when stimulated did show strong frontal activation. These largely anecdotal observations would require additional study to evaluate whether our main observations obtained in the auditory modality from stimulating the aSTP vs. aSTS would also apply to the visual. For instance, would the downstream visual 'object' processing pathway have primarily local temporal lobe inter-connectivity and interact with multisensory regions in the upper bank of the STS to gain functional access to frontal cortex?

Supplementary Tables

Supplementary Table 1. Effects of stimulating left hemisphere aSTP or aSTS sites in M1. Associated with manuscript Fig. 3. Shown are the anatomical sites of significant activation, hemisphere engaged, maximum z-score values of the activated cluster, number of significant voxels in the cluster, and the coordinates of the peak voxel (x, y, z in mm in reference to the macaque brain atlas[2, 3]). (¥) identifies regions significantly activated in the data available for these comparisons in all 3 macaques (M1-3). (ℂ) identifies regions significantly activated in 2 out of 3 of the macaques.

Monkey 1 – Microstimulation of left aSTP and aSTS sites (M1; see manuscript Fig. 3)

aSTP stimulation	Hemi	Max z-score	Num Vox.	xyz (mm)
**Anterior temporal lobe**				
(¥) auditory (anterior ACFs, Table 14)	left	See Table 14	--	--
(¥) aSTS	left	15.92	472	-27 20 9
(¥) temporopolar cortex	left	12.32	174	-16 23 4
(ℂ) temporal area TE	left	6.27	143	-19 20 0
(ℂ) agranular insula	left	12.33	54	-16 23 4
amygdala	left	6.22	56	-14 21 2
hippocampus, entorhinal cortex	left	6.58	25	-14 22 2
**Frontal operculum**				
(ℂ) area PrCo	left	7.89	28	-27 22 10
**aSTS stimulation**	**Hemi**	**Max z-score**	**Num Vox.**	**xyz (mm)**
**Anterior temporal lobe**				
(¥) auditory (anterior ACFs, Table 14)	left	See Table 14	--	--
(¥) aSTS	left	18.24	395	-20 22 -2
(¥) temporopolar cortex	left	19.18	163	-18 22 -2
(¥) hippocampus, entorhinal cortex	left	10.79	131	-14 25 -1
(¥) temporal area TE	left	17.49	355	-23 18 -1
(ℂ) amygdala	left	6.76	137	-10 19 5
(ℂ) agranular insula	left	6.62	75	-18 19 5
**Frontal operculum**				
(ℂ) area PrCo	left	11.18	14	-23 28 5
**Orbital/medial frontal cortex**				
(¥) area 13 (OFC)	left	7.62	101	-14 29 9
**Basal ganglia**				
putamen	left	4.31	24	-11 27 9
**aSTS vs. aSTP stimulation difference**	**Hemi**	**Max z-score**	**Num Vox.**	**xyz (mm)**
**Anterior temporal lobe**				
(¥) auditory (anterior ACFs, Table 14)	left	See Table 14	--	--
(¥) aSTS	left	15.39	127	-20 23 -3
(¥) temporopolar cortex	left	16.32	105	-19 23 -3
(¥) temporal area TE	left	13.10	223	-24 18 -2
**Orbital/medial frontal cortex**				
(ℂ) area 13 (OFC)	left	4.34	13	-14 29 10
area 10 (OFC)	left	3.64	17	-3 46 12
**Other areas**				
pulvinar	left	4.23	11	-7 5 16

Supplementary Table 2. Effects of stimulating left hemisphere aSTP or aSTS sites in M2. Associated with manuscript Fig. 4. Format as in Suppl. Table 1. (¥) identifies regions significantly activated in the data available for these comparisons in all 3 macaques (M1-3). (₵) identifies regions significantly activated in 2 out of 3 of the macaques.

Monkey 2 – Microstimulation of left aSTP and aSTS sites (M2; see manuscript Fig. 4)

**aSTP stimulation**	**Hemi**	**Max z-score**	**Num Vox.**	**xyz(mm)**
**Anterior temporal lobe**				
(¥) auditory (anterior ACFs, see Table 15)	left	See Table 15	--	--
(¥) aSTS	left	6.62	93	-29 15 7
(¥) temporopolar cortex	left	5.85	27	-19 23 1
(₵) temporal area TE	left	6.84	19	-29 14 5
**aSTS stimulation**	**Hemi**	**Max z-score**	**Num Vox.**	**xyz(mm)**
**Anterior temporal lobe**				
(¥) auditory (anterior ACFs, see Table 15)	left	See Table 15	--	--
(¥) aSTS	left	11.08	193	-28 16 7
(¥) temporopolar cortex	left	8.16	55	-17 25 -2
(¥) hippocampus entorhinal/perirhinal	left	4.68	31	-8 10 3
(¥) temporal area TE	left	6.83	46	-29 5 15
(₵) agranular insula	left	7.66	23	-25 16 9
**Orbital/medial frontal cortex**				
(¥) area 13 (OFC)	left	4.93	11	-14 29 8
**aSTS vs. aSTP stimulation difference**	**Hemi**	**Max z-score**	**Num Vox.**	**xyz(mm)**
**Anterior temporal lobe**				
(¥) auditory (anterior ACFs, see Table 15)	left	See Table 15	--	--
(¥) aSTS	left	6.78	76	-25 23 2
(¥) temporopolar cortex	left	5.50	18	-17 26 -2
(¥) temporal area TE	left	5.17	18	-29 14 5
agranular insula	left	7.39	15	-25 15 9
**Orbital/medial frontal cortex**				
(₵) area 14 (OFC/MPFC)	left	3.51	13	-3 33 10

Supplementary Table 3. Effects of stimulating left hemisphere aSTP or aSTS sites in M3. Associated with manuscript Fig. 5. Format as in Suppl. Table 1. (¥) identifies regions significantly activated in the data available for these comparisons in all 3 macaques (M1-3). (₵) identifies regions significantly activated in 2 out of 3 of the macaques.

Monkey 3 – Microstimulation of left aSTP and aSTS sites (M3; see Fig. 5)

**aSTP stimulation**	**Hemi**	**Max z-score**	**Num Voxels**	**xyz (mm)**
**Anterior temporal lobe**				
(¥) auditory (anterior ACFs, see Table 16)	left	See Table 16	--	--
(¥) aSTS	left	6.81	81	-22 25 5
(¥) temporopolar cortex	left	4.53	29	-20 27 3
(₵) agranular insula	left	5.49	30	-26 22 10
**Frontal operculum**				
(₵) area PrCo	left	5.42	33	-22 27 6
dysgranular insula	left	3.92	14	-22 22 10
**aSTS stimulation**	**Hemi**	**Max z-score**	**Num Voxels**	**xyz (mm)**
**Anterior temporal lobe**				
(¥) auditory (anterior ACFs, see Table 16)	left	See Table 16	--	--
(¥) aSTS	left	13.95	285	-18 20 -1
(¥) temporopolar cortex	left	11.93	150	-19 22 -3
(¥) temporal area TE	left	22.20	204	-26 12 5
(¥) hippocampus entorhinal/perirhinal	left	5.72	74	-12 15 3
(₵) amygdala	left	5.89	57	-12 16 2
**Frontal operculum**				
(₵) area PrCo	left	4.46	24	-25 27 10
**Orbital/medial frontal cortex**				
(¥) area 13 (OFC)	left	4.76	54	-13 28 8
area 14 (OFC/MPFC)	left	5.82	160	-1 45 8
area 24 (MPFC)	left	3.94	15	-2 34 16
area 25 (MPFC)	left	4.36	40	-3 34 16
area 32 (MPFC)	left	3.79	29	-2 39 15
**Basal ganglia**				
caudate nucleus	left	5.93	27	-13 16 2
**Other areas**				
visual areas 1-3	left	4.86	338	-12 -7 16
area PGM/31	left	4.25	49	-3 4 33
**aSTS vs. aSTP stimulation difference**	**Hemi**	**Max z-score**	**Num Voxels**	**xyz (mm)**
**Anterior temporal lobe**				
(¥) auditory (anterior ACFs, see Table 16)	left	See Table 16	--	--
agranular insula	left	7.49	16	-21 18 3
(¥) aSTS	left	9.99	340	-18 20 -1
(¥) temporal area TE	left	16.99	166	-26 11 6
(¥) temporopolar cortex	left	7.75	55	-18 22 1
**Orbital/medial frontal cortex**				
(₵) area 13 (OFC)	left	3.50	10	-13 30 10
(₵) area 14 (OFC/MPFC)	left	5.72	55	-1 45 8
area 23 (MFC)	left	4.52	30	-1 11 24
area 25 (MPFC)	left	4.29	19	-6 35 9
**Other areas**				
visual areas 1-3	left	4.09	18	-2 -18 21

Supplementary Table 4. Results from stimulating an anterior belt field. Associated with manuscript Fig. 6. Reported are the anatomical sites of significant activation. Format as in Suppl. Table 1.

Microstimulation of left anterior belt field RTL (M4; see Fig. 6)

Anterior belt field (RTL) stimulation	Hemi	Max z-score	Num Voxels	xyz (mm)
**Anterior temporal lobe**				
auditory (several ACFs, see Table 17)	left/right	See Table 17	--	--
aSTS	left	11.80	397	-29 12 9
temporopolar cortex	left	6.41	36	-20 22 4
temporal area TE	left	7.34	77	-28 12 5
hippocampus entorhinal/perirhinal	left	4.51	34	-14 13 3
granular insula	left	7.87	35	-19 10 17
agranular insula	left	11.76	104	-24 15 8
**Frontal operculum**				
dysgranular insula	left	6.00	16	-23 15 11
area PrCo	left	3.77	8	-23 27 6
**Orbital/medial frontal cortex**				
area 13 (OFC)	left	3.59	19	-11 30 12
**Basal ganglia**				
putamen	left	4.74	87	-15 9 14
caudate nucleus	left	5.03	173	-10 17 22
**Other areas**				
pulvinar	left	4.17	38	-8 11 18
thalamus (several subregions)	left	3.98	130	-3 15 14
reticular thalamic nucleus	left	3.43	16	-11 9 17
parietal area PF, opercular part	left	8.83	54	-26 11 20
somatosensory (primary and secondary)	left	8.88	156	-27 11 19
visual area 2	left	4.97	19	-8 -1 10
cerebellum, anterior lobe	left	4.90	77	-7 -2 11
cerebellum, vermis	left	4.60	22	-4 -6 13
**Right hemisphere areas**				
auditory (koniocortex)	right	4.49	34	21 15 8
aSTS	right	6.07	50	30 17 9
caudate nucleus	right	4.17	89	6 16 22

Supplementary Table 5. Results from stimulating a mid/caudal belt field. Associated with manuscript Fig. 7. Reported are the anatomical sites of significant activation. Format as in Suppl. Table 1.

Microstimulation of left mid/caudal belt field ML (M3; see Fig. 7)

Mid/caudal belt field (ML) stimulation	Hemi	Max z-score	Num Voxels	xyz (mm)
**Temporal lobe**				
auditory (several ACFs, see Table 18)	left/right	See Table 18	--	--
fundus of STS	left	5.71	23	-16 5 10
hippocampus entorhinal/perirhinal	left	5.51	32	-15 1 10
agranular insula	left	20.65	32	-16 14 12
granular insula	left	15.55	57	-16 13 13
**Frontal operculum**				
dysgranular insula	left	20.65	5	-16 14 12
**Ventral/dorsal prefrontal cortex**				
area 44	left	8.09	28	-17 30 18
area 45	left	13.93	60	-19 31 20
area 46	left	6.05	11	-16 30 21
area 8	left	12.35	119	-22 30 21
**Basal ganglia**				
external globus pallidus	left	16.50	48	-13 12 14
caudate nucleus	left	6.37	69	-11 12 20
internal globus pallidus	left	4.87	12	-11 13 9
putamen	left	20.82	321	-16 14 12
**Other areas**				
area 3b (somatosensory)	left	5.23	11	-27 18 16
somatosensory primary and secondary	left	7.71	22	-26 18 15
pulvinar	left	12.67	53	-5 7 14
thalamus (several subregions)	left	11.47	140	-5 9 12
external medullary lamina	left	7.10	11	-14 6 15
reticular thalamic nucleus	left	10.71	101	-15 8 14
superior colliculus	left	9.25	21	-5 6 12
inferior colliculus	left	5.53	10	-6 4 10
periaqueductal gray	left	5.53	20	-3 6 12
Dorsal lateral geniculate	left	13.53	34	-14 11 11
**Right hemisphere areas**				
area 4 (motor)	right	8.33	17	22 20 25
area 3a (somatosensory)	right	4.50	46	22 18 24
putamen	right	8.28	83	16 15 10

Supplementary Table 6. Results from stimulating frontal cortex area 45. Associated with manuscript Fig. 8. Format as in Suppl. Table 1. See Suppl. Table 19 for greater specificity in the ACFs that were significantly activated.

Microstimulation of left area 45 (M4; see manuscript Fig. 8)

Area 45 stimulation	Hemi	Max z-score	Num Voxels	xyz (mm)
**Anterior temporal lobe**				
auditory (anterior ACF, see Table 19)	left	Table 19	--	--
**Frontal operculum**				
area PrCo	left	7.98	28	-24 31 9
**OFC**				
area 13	left	5.35	18	-15 34 16
**Ventro-lateral PFC**				
area 44	left	18.01	163	-18 31 16
area 45	left	18.28	159	-18 32 15
**Dorso-lateral PFC**				
area 6	left	7.32	275	-22 31 12
area 8	left	12.21	134	-16 25 19
area 9	left	3.84	17	-5 38 25
**Basal ganglia**				
caudate nucleus	left	7.82	181	-6 24 17
putamen	left	5.31	167	-9 25 12
**Other areas**				
area 4 (motor)	left	4.18	17	-17 17 32
pulvinar	left	3.92	12	-4 8 15
thalamus (several subregions)	left	8.60	218	-2 10 16
reticular thalamic nucleus	left	5.69	10	-5 16 16
superior colliculus	left	5.85	33	-3 6 17
periaqueductal gray	left	8.48	27	-2 7 15
cerebellum, anterior lobe	left	3.68	23	-4 -1 7
**Right hemisphere**				
area 44	right	8.86	38	19 32 16
area 45	right	10.96	54	19 33 15

Supplementary Table 7. Results from stimulating frontal cortex area 6va/F5. Associated with manuscript Fig. 9. Format as in Suppl. Table 1. See Suppl. Table 20 for greater specificity in the ACFs that were significantly activated.

Microstimulation of left area 6va/F5 (M3; see manuscript Fig. 9)

Area 6va stimulation	Hemi	Max z-score	Num Voxels	xyz (mm)
**Temporal lobe**				
auditory (belt/parabelt ACFs, see Table 20)	left	Table 20	--	--
STS	left	7.14	27	-25 21 11
temporal area TE	left	4.76	60	-27 10 8
**Frontal operculum**				
area PrCo	left	17.60	69	-23 27 12
dysgranular insula	left	3.44	11	-19 24 15
**Ventro-lateral PFC**				
area 44	left	15.03	85	-24 29 11
area 45	left	7.44	54	-23 31 19
**Basal ganglia**				
putamen	left	5.01	134	-10 25 14
**Other areas**				
area 3a/b (somatosensory)	left	15.88	132	-24 26 10
gustatory cortex	left	18.23	38	-22 28 11

Supplementary Table 8. Results from stimulating frontal operculum. Associated with manuscript Fig. 10. Format as in Suppl. Table 1. See Suppl. Table 21 for greater specificity in the ACFs that were significantly activated.

Microstimulation of left frontal operculum (M3; see manuscript Fig. 10)

Frontal operculum stimulation	Hemi	Max z-score	Num Voxels	xyz (mm)
**Anterior temporal lobe**				
auditory (several ACFs, see Table 21)	left	Table 21	--	--
aSTS	left	9.24	191	-16 20 -4
temporopolar cortex	left	9.58	124	-19 21 5
temporal area TE	left	10.98	141	-18 19 -3
hippocampus, entorhinal cortex	left	19.74	288	-7 8 2
agranular insula	left	13.64	376	-17 27 10
amygdala	left	16.00	195	-7 18 4
**Frontal operculum**				
area ProM (promotor)	left	15.01	53	-22 29 8
dysgranular insula	left	13.02	75	-21 19 13
**Orbital/medial frontal cortex**				
area 12 (OFC)	left	12.29	173	-21 30 8
area 13 (OFC)	left	9.23	42	-14 29 8
area 23 (MFC)	left	4.16	30	-1 15 25
**Ventro-lateral PFC**				
area 44	left	9.89	65	-19 26 14
area 45	left	4.11	16	-16 32 15
**Basal ganglia**				
putamen	left	6.49	269	-12 14 7
caudate nucleus	left	9.28	151	-11 15 3
**Other areas**				
periaqueductal gray	left	5.26	24	-1 8 15
nucleus accumbens	left	8.28	51	-6 25 8
reticular thalamic nucleus	left	7.19	34	-16 7 10
area 3a/b (somatosensory)	left	8.41	103	-19 26 14
visual area 2	left	4.36	23	-11 -3 13
cerebellum, deep nuclei	left	7.20	20	-9 -8 4
cerebellum	left	9.79	105	-11 -7 4

Supplementary Table 9. Summary anatomical areas activated with left hemisphere aSTP microstimulation in M1. Associated with Suppl. Fig. 5. Format as in Suppl. Table 1.

Left hemisphere aSTP stimulation	Hemi	Max z-score	Num Voxels	xyz (mm)
**Anterior temporal lobe**				
auditory (several ACFs)	left	Table 14	--	--
aSTS	left	13.27	287	-21 24 6
temporopolar cortex	left	12.75	57	-15 22 3
temporal area TE	left	4.64	24	-20 17 1
agranular insula	left	9.83	31	-21 24 7
**Other areas**				
visual areas 1-3	left/right	3.90	373	(-)16 -10 21

Supplementary Table 10. Summary anatomical areas activated with right hemisphere aSTP microstimulation in M1. Associated with Suppl. Fig. 5. Format as in Suppl. Table 1.

Right hemisphere aSTP stimulation	Hemi	Max z-score	Num Voxels	xyz (mm)
**Anterior temporal lobe**				
auditory (several ACFs)	right	Table 13	--	--
aSTS	right	19.00	322	25 23 10
temporopolar cortex	right	15.27	91	19 23 4
agranular insula	right	12.96	72	19 22 4
**Basal ganglia**				
caudate nucleus / putamen	right	6.70	62	17 13 7

Supplementary Table 11. Summary anatomical areas activated with right hemisphere stimulation of aSTP or aSTS sites in M1. Associated with Suppl. Fig. 6. Format as in Suppl. Table 1.

Stimulating right hemisphere aSTP and aSTS (M1)

aSTP stimulation (RH)	Hemi	Max z-score	Num Voxels	xyz (mm)
**Anterior temporal lobe**				
auditory (anterior ACFs, Table 13)	right	Table 13	--	--
aSTS	right	26.15	488	25 26 8
temporopolar cortex	right	21.41	225	18 23 2
temporal area TE	right	9.01	154	27 14 5
hippocampus entorhinal/perirhinal	right	9.27	170	13 23 5
amygdala	right	9.50	149	15 18 1
agranular insula	right	15.45	71	24 22 9
**Frontal operculum**				
area PrCo	right	23.33	105	24 27 7
dysgranular insula	right	4.99	16	17 18 9
**Orbital/medial frontal cortex**				
area 13 (OFC)	right	4.32	11	12 29 10
**aSTS stimulation (RH)**	**Hemi**	**Max z-score**	**Num Voxels**	**xyz (mm)**
**Anterior temporal lobe**				
auditory (anterior ACFs, Table 13)	right	Table 13	--	--
aSTS	right	28.19	525	21 25 3
temporopolar cortex	right	28.46	266	19 26 -1
temporal area TE	right	24.19	200	26 18 1
hippocampus entorhinal/perirhinal	right	18.79	254	13 26 -1
amygdala	right	16.07	257	8 20 5
agranular insula	right	20.39	133	18 22 5
**Fronto-temporal operculum**				
area PrCo	right	26.24	71	24 28 6
dysgranular insula	right	5.56	26	20 17 10
**Basal ganglia**				
nucleus accumbens	right	9.17	64	3 28 11
caudate nucleus	right	8.97	181	3 27 14
putamen	right	8.09	68	18 18 9
**Orbital/medial frontal cortex**				
area 10 (MPFC/OFC)	right	7.36	177	1 46 14
area 11 (OFC)	right	5.52	18	4 39 15
areas 12, 13 (OFC)	right	14.99	328	12 30 10
area 14 (MPFC)	right	17.62	223	1 43 14
area 10 (MPFC/OFC)	left	7.08	14	-2 45 14
area 14 (MPFC)	left	16.55	125	-1 42 14
area 25 (MPFC)	left	14.72	76	-2 34 11
area 32 MPFC	left	7.08	27	-2 40 17
**aSTS vs aSTP stimulation difference**	**Hemi**	**Max z-score**	**Num Voxels**	**xyz (mm)**
**Anterior Temporal Lobe**				
auditory (anterior ACFs, Table 13)	right	Table 13	--	--
aSTS	right	18.60	287	21 26 -1
temporopolar cortex	right	17.69	188	21 26 -1
temporal area TE	right	16.91	97	21 21 -2
hippocampus entorhinal/perirhinal	right	11.02	136	13 24 -2
amygdala	right	8.07	173	8 19 5
agranular insula	right	12.05	154	20 20 7
**Basal ganglia**				
nucleus accumbens	right	8.09	48	3 28 11
caudate nucleus	right	7.29	162	2 25 17
putamen	right	5.52	37	12 28 11
**Frontal operculum**				
area PrCo	right	8.58	7	24 28 6
dysgranular insula	right	4.22	3	20 17 10
**Orbital/medial frontal cortex**				
area 10 (MPFC/OFC)	right	11.37	276	1 42 15
area 11 (OFC)	right	4.61	16	4 39 15
areas 12 and 13 (OFC)	right	8.50	153	12 29 10
area 14 (OFC/MPFC)	right	8.50	102	2 28 11
area 25 (MPFC)	right	12.09	210	1 33 12
area 32 (MPFC)	right	6.58	41	1 40 16
areas 11 (OFC)	left	4.01	23	-8 37 17
area 14 (OFC/MPFC)	left	10.38	92	-2 43 14
area 25 (MPFC)	left	10.14	54	-2 33 12
area 32 (MPFC)	left	5.08	13	-1 40 17

Supplementary Table 12. Summary anatomical areas activated by microstimulating the aSTS in M4. Associated with Suppl. Fig. 7. Format as in Suppl. Table 1.

Microstimulation of aSTS in M4	Hemi	Max z-score	Num Voxels	xyz (mm)
**Anterior temporal lobe**				
aSTS	left	11.43	118	-20 15 4
temporopolar cortex	left	3.53	4	-19 22 -1
temporal area TE	left	15.16	121	-23 17 2
**Orbital frontal cortex**				
area 12 (OFC)	left	5.26	33	-16 36 14

Supplementary Table 13. Summary ACFs activated by microstimulating the right hemisphere aSTP in M1. Associated with Supplementary Figure 5 and 8. Format as in Suppl. Table 1.

M1 – Microstimulating right hemisphere sites (Suppl Figs. 5 and 8)

M1 – Right aSTP stimulation	Hemi	Max z-score	Num Voxels	xyz (mm)
Ts2	right	25.04	216	21 24 4
Ts1	right	23.21	106	20 25 3
Pro	right	15.49	92	18 27 1
RTL	right	22.16	38	25 21 10
RPB	right	21.36	78	25 23 8
RT	right	14.53	32	23 20 10
RTM	right	4.46	22	20 20 8
AL	right	15.42	37	25 19 12
R	right	8.52	24	23 19 12
A1	right	6.83	11	23 14 16
**M1 – Right aSTS stimulation**	**Hemi**	**Max z-score**	**Num Voxels**	**xyz (mm)**
Ts2	right	28.19	232	21 25 3
Ts1	right	27.03	107	20 25 3
Pro	right	27.31	122	18 26 -1
RTL	right	14.82	38	25 21 10
RPB	right	15.81	83	25 23 8
RT	right	16.94	41	20 19 8
RTM	right	17.74	29	20 20 8
MM	right	5.56	12	20 17 10
AL	right	10.03	46	25 19 12
R	right	7.47	51	22 18 9
A1	right	3.28	7	22 15 15
**M1 - Contrast aSTS-aSTP**	**Hemi**	**Max z-score**	**Num Voxels**	**xyz (mm)**
Ts2	right	12.90	188	19 21 6
Ts1	right	9.60	66	19 23 2
Pro	right	14.70	52	17 26 -1
RPB	right	4.44	5	27 17 7
RTM	right	11.54	29	20 20 8
RT	right	10.56	18	20 19 8
MM	right	4.22	2	20 17 10
R	right	4.68	5	22 18 9
**M1 - Contrast aSTP-aSTS**	**Hemi**	**Max z-score**	**Num Voxels**	**xyz (mm)**
RTL	right	6.88	13	25 21 10
RPB	right	5.59	6	25 22 9

Supplementary Table 14. Summary ACFs activated by microstimulating the left hemisphere aSTP in M1. Associated with Supplementary Figure 5 and 8. Format as in Suppl. Table 1.

M1 – Microstimulating left hemisphere sites (Suppl Figs. 5-8)

M1 - Microstimulate aSTP	Hemi	Max z-score	Num Voxels	xyz (mm)
Ts2	left	13.51	154	-17 21 3
Ts1	left	12.02	84	-21 24 3
Pro	left	12.10	77	-16 23 3
RPB	left	15.92	65	-27 20 9
RTL	left	14.86	25	-25 20 9
RT	left	10.87	37	-21 19 4
RTM	left	11.76	16	-19 20 4
AL	left	8.58	10	-26 18 10
**M1 - Microstimulate aSTS**	**Hemi**	**Max z-score**	**Num Voxels**	**xyz (mm)**
Ts2	left	11.60	94	-22 25 2
Ts1	left	15.51	67	-19 25 -2
Pro	left	14.63	81	-18 25 -2
RPB	left	4.86	21	-27 21 8
RTM	left	6.85	9	-18 19 4
RTL	left	3.49	11	-25 21 8
RT	left	7.43	9	-23 19 4
**M1 - Contrast aSTS-aSTP**	**Hemi**	**Max z-score**	**Num Voxels**	**xyz (mm)**
Ts1	left	6.37	15	-23 22 1
Pro	left	12.52	80	-19 25 -2

M1 - Contrast aSTP-aSTS	Hemi	Max z-score	Num Voxels	xyz (mm)
Ts2	left	9.09	74	-27 20 9
Ts1	left	8.67	85	-27 21 8
Pro	left	5.38	43	-21 24 3

Supplementary Table 15. Summary ACFs activated by microstimulating the left hemisphere aSTP in M2.

Associated with manuscript Fig. 4. Format as in Suppl. Table 1.

M2 – Microstimulating left hemisphere sites (manuscript Fig. 4)

M2 - Microstimulate aSTP	Hemi	Max z-score	Num Voxels	xyz (mm)
Ts2	left	6.62	32	-29 15 7
Ts1	left	5.23	38	-23 24 1
Pro	left	6.25	56	-19 24 1
**M2 - Microstimulate aSTS**	**Hemi**	**Max z-score**	**Num Voxels**	**xyz (mm)**
Ts2	left	11.08	74	-28 16 7
Ts1	left	10.12	49	-24 22 1
Pro	left	9.18	63	-21 24 -2
RPB	left	7.05	44	-29 14 9
RTL	left	5.82	14	-29 12 14
RT	left	5.83	22	-26 13 9
CPB	left	3.41	7	-29 8 17
**M2 - Contrast aSTS-aSTP**	**Hemi**	**Max z-score**	**Num Voxels**	**xyz (mm)**
Ts2	left	7.39	56	-25 15 9
Ts1	left	6.78	40	-25 23 2
Pro	left	5.98	22	-16 26 -2
RPB	left	5.03	14	-27 15 9
RTL	left	3.53	2	-29 12 13
RT	left	3.80	6	-24 14 11
**M2 - Contrast aSTP-aSTS**	**Hemi**	**Max z-score**	**Num Voxels**	**xyz (mm)**
Ts1	left	5.99	15	-20 23 3
Pro	left	7.02	23	-20 24 3

Supplementary Table 16. Summary ACFs activated by microstimulating the left hemisphere aSTP in M3.

Associated with manuscript Fig. 5. Format as in Suppl. Table 1.

M3 – Microstimulating left hemisphere sites (manuscript Fig. 5)

M3 - Microstimulate aSTP	Hemi	Max z-score	Num Voxels	xyz (mm)
Ts2	left	3.79	13	-24 18 10
Ts1	left	6.81	54	-22 25 5
Pro	left	6.12	49	-21 25 4
**M3 - Microstimulate aSTS**	**Hemi**	**Max z-score**	**Num Voxels**	**xyz (mm)**
Ts2	left	6.02	33	-25 18 7
Ts1	left	8.21	93	-21 19 3
Pro	left	10.43	155	-18 21 1
**M3 - Contrast aSTS-aSTP**	**Hemi**	**Max z-score**	**Num Voxels**	**xyz (mm)**
Ts2	left	4.12	6	-24 19 6
Ts1	left	5.68	25	-20 20 2
Pro	left	7.75	73	-18 22 1
R	left	3.92	4	-24 8 14
A1	left	3.88	3	-24 7 15
**M3 - Contrast aSTP-aSTS**	**Hemi**	**Max z-score**	**Num Voxels**	**xyz (mm)**
Ts1	left	3.42	2	-27 22 7

Supplementary Table 17. Summary ACFs activated by stimulating an anterior lateral belt field. Associated with manuscript Fig. 6. Format as in Suppl. Table 1.

M4 - Stimulating anterior belt (RTL)	Hemi	Max z-score	Num Voxels	xyz (mm)
Ts2	left	11.76	152	-24 15 8
Ts1	left	10.95	192	-28 18 6
Pro	left	7.82	99	-21 16 7
RPB	left	11.96	66	-29 14 10
RTL	left	8.48	26	-28 14 13
RTM	left	11.40	44	-22 11 12
RT	left	13.01	75	-23 12 11
R	left	10.02	87	-22 10 14
AL	left	8.17	16	-27 11 16
MM	left	9.10	41	-21 9 14
ML	left	8.69	16	-27 11 20
A1	left	9.04	127	-24 6 21
CL	left	5.89	33	-25 5 23
CPB	left	6.54	46	-26 3 21
Ts2	right	7.39	41	23 20 7
Ts1	right	3.63	15	26 24 5
Pro	right	4.68	4	22 19 7
RPB	right	5.35	10	29 17 9
RTM	right	4.05	12	23 15 11
RT	right	3.99	24	24 14 12
MM	right	3.58	5	23 14 13

Supplementary Table 18. Summary ACFs activated by stimulating a mid/caudal lateral belt field. Associated with manuscript Fig. 7. Format as in Suppl. Table 1.

M3 - Stimulating mid/caudal belt (ML)	Hemi	Max z-score	Num Voxels	xyz (mm)
Ts2	left	4.42	2	-16 20 7
Pro	left	20.61	61	-16 15 11
RTM	left	8.55	4	-16 18 8
R	left	9.70	8	-18 15 11
A1	left	12.81	34	-16 11 15
MM	left	20.50	18	-16 15 11
CM	left	6.64	20	-13 7 18
Pro	right	7.51	36	17 15 10
MM	right	5.28	3	18 14 11

Supplementary Table 19. Summary ACFs activated by stimulating frontal cortex area 45. Associated with manuscript Fig. 8. Format as in Suppl. Table 1.

M4 - Stimulate area 45	Hemi	Max z-score	Num Voxels	xyz (mm)
Pro	left	4.32	10	-14 22 9

Supplementary Table 20. Summary ACFs activated by stimulating frontal cortex area 6va/F5. Associated with manuscript Fig. 9. Format as in Suppl. Table 1.

M3- Stimulate area 6va/F5	Hemi	Max z-score	Num Voxels	xyz (mm)
Pro	left	3.87	20	-14 19 9
RPB	left	3.43	11	-27 15 13

Supplementary Table 21. Summary ACFs activated by stimulating frontal operculum. Associated with manuscript Fig. 10. Format as in Suppl. Table 1.

M3 - Stimulate operculum (insula)	Hemi	Max z-score	Num Voxels	xyz (mm)
Ts2	left	9.58	102	-19 21 5
Ts1	left	7.79	55	-17 24 3
Pro	left	10.31	112	-14 25 5
RTL	left	4.86	6	-24 20 6
RTM	left	9.57	17	-19 21 5
RT	left	9.07	32	-19 20 9
R	left	12.89	73	-20 19 10
MM	left	7.97	18	-18 18 8
A1	left	3.98	5	-15 7 16

Supplementary Table 22. Summary anatomical areas activated by microstimulating a face-sensitive cluster in the fundus of the STS. Associated with Suppl. Fig. 10. Format as in Suppl. Table 1.

**Stimulating fSTS**	**Hemi**	**Max z-score**	**Num Voxels**	**xyz (mm)**
**Temporal-parietal areas**				
hippocampus entorhinal/perirhinal	left	15.69	31	-18 10 4
STS	left	17.45	372	-20 17 2
middle temporal area (vis. area 5)	**right**	3.86	28	14 -5 28
parieto-occipital area	left	4.42	17	-4 -11 33
temporal area TE	left	20.04	301	-24 13 5
temporal area TF	left	11.19	44	-20 6 4
temporopolar cortex	left	7.63	49	-17 22 -3
visual areas 1-4	**left/right**	4.56	824	8/-8 -13 34
LOP/LIP	left	3.34	18	-10 -10 32
fundus of STS	left	18.69	97	-21 5 8
area 23	left	3.71	39	-1 13 24
**Basal ganglia**				
caudate nucleus	left	5.18	16	-16 14 4
**Cerebellum**				
cerebellum, several areas	left	4.19	375	-6 -5 12

Supplementary Discussion

Why Remifentanil Anesthesia and the impact on the BOLD response

The main reason for using opiates is the fact that they selectively act on the pain matrix while largely sparing sensory areas. The concentration of opiate receptors, i.e. μ, κ, σ and δ[4], varies across different brain regions[5], with high concentrations mainly found in the so-called pain matrix[6]. The latter refers to the regions commonly activated in response to noxious stimulation, namely thalamus, somatosensory/motor cortices, insula, cingulate cortex, basal ganglia, as well as certain areas of the frontal and parietal cortices (medial areas in particular). Remifentanil, an ultra-fast-acting μ-opioid receptor agonist, has no significant effect on the neurovascular activity of brain areas that do not belong to the pain matrix. The visual cortex in particular does not bind remifentanil and the extent to which auditory cortex does is not clear owing to its close proximity to somatosensory cortex which does bind reminfentanil[7].

Region-specific effects of remifentanil have been studied by means of electrocorticography (ECoG) and fMRI. ECoG recordings showed no significant effects of remifentanil on spike rate, and in epileptogenic zones remifentanil may even increase spiking activity[8]. FMRI studies typically demonstrate remifentanil-induced BOLD activations that have been attributed to the inhibition of GABA-ergic neurons[9]. Note that fMRI studies showed that while the activation of the pain matrix by pain stimuli depends on the remifentanil dose, the activation of visual cortex by a visual stimulus remains the same for all doses of remifentanil[10-13]. In a similar fashion, remifentanil does not modulate cerebrovascular CO_2 reactivity to hypercapnic stimulation, even in areas with high opioid receptor densities[11], and it only causes mild reductions of the positive BOLD response in the primary sensorimotor cortices, bilateral extrastriate visual cortex, and much of auditory cortex, insula, caudate nucleus and inferior temporal gyrus.

In conclusion, auditory and visual information processing, including much of association cortex and ventral frontal cortex, appears to be largely preserved during remifentanil anesthesia, while regions belonging to the pain matrix (including parts of medial frontal and parietal cortex) are affected to various degrees. Supporting the aforementioned pharmacological, ECoG and fMRI findings from different laboratories, including our macaque physiology and fMRI experiments show that remifentanil only mildly affects the magnitude and time course of neural and vascular responses[14-18]. The paper by Goense and Logothetis in particular provides a detailed comparison between physiological and MRI signals: barring small differences in amplitude and temporal profile (signals in the anesthetized animal may often have stronger transients than in the unanesthetized preparation), both signals are shown to be unaffected by anesthesia, as is the relationship of the BOLD signal to the underlying neural activity[15].

Supplementary References

1. Moeller, S., Freiwald, W.A. & Tsao, D.Y. Patches with links: a unified system for processing faces in the macaque temporal lobe. *Science* **320**, 1355-1359 (2008).

2. Saleem, K.S. & Logothetis, N.K. *A Combined MRI and Histology: Atlas of the Rhesus Monkey Brain in Stereotaxic Coordinates* (Academic Press, London, 2007).

3. McLaren, D.G., *et al.* A population-average MRI-based atlas collection of the rhesus macaque. *Neuroimage* **45**, 52-59 (2009).

4. Pfeiffer, A., Pasi, A., Mehraein, P. & Herz, A. Opiate receptor binding sites in human brain. *Brain Research* **248**, 87-96 (1982).

5. Leppä, M., *et al.* Acute opioid effects on human brain as revealed by functional magnetic resonance imaging. *Neuroimage* **31**, 661-669 (2006).

6. Talbot, J.D., *et al.* Multiple representations of pain in human cerebral cortex. *Science* (1991).

7. Jones, A.K.P., *et al.* In vivo distribution of opioid receptors in man in relation to the cortical projections of the medial and lateral pain systems measured with positron emission tomography. *Neuroscience letters* **126**, 25-28 (1991).

8. Grønlykke, L., *et al.* Remifentanil-induced spike activity as a diagnostic tool in epilepsy surgery. *Acta Neurologica Scandinavica* **117**, 90-93 (2008).

9. Zieglgansberger, W., French, E.D., Siggins, G.R. & Bloom, F.E. Opioid peptides may excite hippocampal pyramidal neurons by inhibiting adjacent inhibitory interneurons. *Science* **205**, 415-417 (1979).

10. Iannetti, G.D., *et al.* Pharmacological modulation of pain-related brain activity during normal and central sensitization states in humans. *Proceedings of the National Academy of Sciences of the United States of America* **102**, 18195-18200 (2005).

11. Pattinson, K.T.S., Rogers, R., Mayhew, S.D., Tracey, I. & Wise, R.G. Pharmacological FMRI: measuring opioid effects on the BOLD response to hypercapnia. *Journal of Cerebral Blood Flow & Metabolism* **27**, 414-423 (2007).

12. Wise, R.G., *et al.* Combining fMRI with a pharmacokinetic model to determine which brain areas activated by painful stimulation are specifically modulated by remifentanil. *Neuroimage* **16**, 999-1014 (2002).

13. Rieck, A., *et al.* Functional imaging of the visual cortex during wakefulness and during intravenous anaesthesia: A-6. *European Journal of Anaesthesiology (EJA)* **22**, 2 (2005).

14. Logothetis, N.K., Guggenberger, H., Peled, S. & Pauls, J. Functional imaging of the monkey brain. *Nat Neurosci* **2**, 555-562 (1999).

15. Goense, J. & Logothetis, N.K. Neurophysiology of the BOLD fMRI signal in awake monkeys. *Current Biology* **18**, 631-640 (2008).

16. Logothetis, N.K., Pauls, J., Augath, M., Trinath, T. & Oeltermann, A. Neurophysiological investigation of the basis of the fMRI signal. *Nature* **412**, 150-157 (2001).

17. Zappe, A.C., Uludağ, K., Oeltermann, A., Uğurbil, K. & Logothetis, N.K. The influence of moderate hypercapnia on neural activity in the anesthetized nonhuman primate. *Cerebral Cortex* **18**, 2666-2673 (2008).

18. Zappe, A.C., Uludağ, K. & Logothetis, N.K. Direct measurement of oxygen extraction with fMRI using 6% CO2 inhalation. *Magnetic resonance imaging* **26**, 961-967 (2008).
